# Protein-Protein Interactions of Tandem Affinity Purified Protein Kinases from Rice

**DOI:** 10.1371/journal.pone.0006685

**Published:** 2009-08-19

**Authors:** Jai S. Rohila, Mei Chen, Shuo Chen, Johann Chen, Ronald L. Cerny, Christopher Dardick, Patrick Canlas, Hiroaki Fujii, Michael Gribskov, Siddhartha Kanrar, Lucas Knoflicek, Becky Stevenson, Mingtang Xie, Xia Xu, Xianwu Zheng, Jian-Kang Zhu, Pamela Ronald, Michael E. Fromm

**Affiliations:** 1 Plant Science Initiative, University of Nebraska, Lincoln, Nebraska, United States of America; 2 Department of Chemistry, University of Nebraska, Lincoln, Nebraska, United States of America; 3 Department of Plant Pathology, University of California Davis, Davis, California, United States of America; 4 Department of Biological Sciences, Purdue University, West Lafayette, Indiana, United States of America; 5 Botany & Plant Sciences, University of California Riverside, Riverside, California, United States of America; GSF Research Center for Environment and Health, Germany

## Abstract

Eighty-eight rice (*Oryza sativa*) cDNAs encoding rice leaf expressed protein kinases (PKs) were fused to a Tandem Affinity Purification tag (TAP-tag) and expressed in transgenic rice plants. The TAP-tagged PKs and interacting proteins were purified from the T1 progeny of the transgenic rice plants and identified by tandem mass spectrometry. Forty-five TAP-tagged PKs were recovered in this study and thirteen of these were found to interact with other rice proteins with a high probability score. *In vivo* phosphorylated sites were found for three of the PKs. A comparison of the TAP-tagged data from a combined analysis of 129 TAP-tagged rice protein kinases with a concurrent screen using yeast two hybrid methods identified an evolutionarily new rice protein that interacts with the well conserved cell division cycle 2 (CDC2) protein complex.

## Introduction

The recently revealed sequences of many plant genomes highlight the fact that most of the cell's biological complexity occurs at the level of protein structure, protein interactions, and post-translational modifications, collectively defined herein as the proteome [1]. Environmental stresses are signaled in large part through changes in the proteome and metabolome. These signals are important for the plant's stress responses, particularly to water deficits and disease as these are the most important factors in determining plant yields and quality [2]. Protein kinases (PKs) and phosphatases are critical components of many plant stress signaling pathways including those for cold, drought and salt tolerance [3], [4], [5], pathogen recognition for disease resistance [6], [7], ABA [8], ethylene signaling [9], regulation of carbon metabolism [10] and cell cycle regulation [11]. Signaling specificity is often determined by the scaffolding, anchoring and/or adaptor proteins that organize the regulatory proteins [12], [13], [14], [15]. Determining these protein-protein interactions is important for developing an understanding of the mechanisms that PKs use to recognize their substrates and mediate signaling specificity.

Rice has become a model for cereal genomics [16] in large part because its small 389 Mb genome has been sequenced [17], [18]. A sequenced genome is essential for proteomic methods requiring interpretation of mass spectrometry (MS) data for the identification of peptides and the corresponding protein they were derived from. Tandem Affinity Purification (TAP) is a MS-based approach [19] for identifying interacting proteins that are co-purified after the gene for a target protein is fused to a tandem affinity protein tag (TAP-tag). The TAP-tagged protein and any associated proteins are then isolated from the host organism in two sequential affinity purification steps. This TAP-tagging method has been used to identify protein complexes from yeast [20], insect [21], human cells [22] and plants [23], [24], [25], [26].

In order to discover new protein interactions in PK signaling networks in cereal leaves, we have TAP-tagged 88 rice leaf-expressed PKs for subsequent MS analysis of purified protein complexes isolated from transgenic rice plants. We report here that forty-five of the eighty-eight TAP-tagged PKs can be purified in amounts sufficient for MS analysis, and thirteen of these have been isolated as complexes with one or more interacting proteins. The authenticity of some of the interacting proteins in the isolated protein complexes is supported by evidence from interactions of homologous proteins in other organisms as well as by similar interactions by related family members. An evolutionarily new protein unique to rice that is part of a protein complex that regulates cell division has been identified by comparison of TAP-tag-derived and yeast two hybrid screening data [27].

## Results

### Proteins Interacting with TAP-tagged PKs

Forty-five TAP-tagged PKs were identified by two or more peptides after purification and MS/MS analysis (Supplementary Table S1). The criteria for identifying a protein interacting with these TAP-tagged PKs were the identification of two independent peptides from a protein with individual molecular weight search (MOWSE) scores of at least the identity level and that the identified protein was present in less than 5% of the purifications [28]. Thirteen TAP-tagged PKs showing protein interactions meeting these criteria are shown in Table 1 and are discussed below. Supplementary Table S1-A contains additional possible interacting proteins for these thirteen purifications where either one peptide with a good MOWSE score was identified or peptides were from an abundant protein present in less than 5% of the purifications. Analyses of TAP-tagged PKs that recovered only a single peptide for potential interacting proteins or multiple peptides for unique isoform members of a family of abundant proteins are shown in Supplemental Table S1-B. Single peptide identifications would require further verification to distinguish them from potential contaminants.

**Table 1:** TAP-tagged protein kinases and interacting proteins.

#	Protein Name	TIGR_ID	% coverage	No. of peptides	Score
**1**	**Calcium-dependent protein kinase, isoform 11, putative, expressed**	Os03g03660	20	11	789
	Serine hydroxymethyltransferase, mitochondrial precursor, putative, expressed	Os03g52840	21	6	450
	Nascent polypeptide-associated complex alpha subunit-like protein 3, putative, expressed	Os01g71230	12	2	158
**2**	**Lectin receptor kinase 7, putative, expressed**	Os07g38800	26	10	722
	lectin-like receptor kinase 7, putative	Os02g19530	3	2	89
	Actin-7, putative, expressed	Os11g06390	19	5	347
	IAP100, putative, expressed	Os10g35030	8	2	178
**3**	**Lectin protein kinase, putative, expressed**	Os07g38810	26	9	1158
	Cysteine protease 1 precursor, putative, expressed	Os04g57440	6	2	157
**4**	**Protein kinase domain containing protein**	Os01g14510	22	5	375
	Calcium-dependent protein kinase, isoform 1, putative, expressed	Os03g03660	4	3	182
	IAP100, putative, expressed	Os10g35030	8	2	159
	Salt stress-induced protein, putative, expressed	Os01g24710	18	2	120
**5**	**Protein kinase APK1B, chloroplast precursor, putative, expressed**	Os03g06330	70	11	1560
	DEAD/DEAH box helicase family protein, expressed	Os03g61220	3	2	151
**6**	**Serine/threonine-protein kinase RLCKVII**	Os07g49470	30	6	594
	HEAT repeat family protein, karyopherin-beta 3 variant expressed	Os07g38760	6	3	336
	HEAT repeat family protein, expressed	Os03g49420	6	4	220
**7**	**Protein kinase APK1A, chloroplast precursor**	Os05g02020	66	10	2016
	Dynamin-2A, putative, expressed	Os06g13820	24	14	1210
	Dynamin-2B, putative, expressed	Os02g50550	18	13	816
	DEAD/DEAH box helicase family protein, expressed	Os03g61220	23	8	1056
	Dynamin-related protein 1A, putative, expressed	Os05g48240	19	7	819
	Dynamin-related protein 1C, putative, expressed	Os03g50520	22	7	584
	Dynamin-related protein 1C, putative, expressed	Os10g41820	18	8	498
	DEAD/DEAH box helicase family protein, expressed	Os01g43120	16	6	386
	pentatricopeptide, putative, expressed	Os03g63910	4	2	97
	linker histone H1 and H5 family protein, expressed	Os03g58470	15	2	154
	expressed protein	Os02g22070	9	2	125
	Ribonuclease T2 family protein, expressed	Os09g36700	7	1	62
**8**	**Protein kinase domain containing protein, expressed**	Os06g50100	37	8	845
	Heat shock cognate 70 kD protein, putative, expressed	Os01g62290	12	5	360
**9**	**Protein kinase domain containing protein, expressed**	Os01g67340	51	8	1167
	Actin-1, putative, expressed	Os12g44350	7	2	116
	Transketolase, chloroplast precursor, putative, expressed	Os06g04270	5	2	156
	Triosephosphate isomerase, chloroplast precursor, putative, expressed	Os09g36450	14	2	145
	2-cys peroxiredoxin BAS1, chloroplast precursor, putative, expressed	Os02g33450	17	3	177
	CBS domain containing protein, expressed	Os03g52690	14	2	117
**10**	**WAK-like kinase, putative, expressed**	Os03g12470	17	9	616
	Cysteine protease 1 precursor, putative, expressed	Os04g57440	6	2	139
**11**	**Carbon catabolite derepressing protein kinase, putative, expressed**	Os08g37800	35	13	1056
	Protein kinase AKINbetagamma-2, putative, expressed	Os03g63940	32	7	1070
	SNF4, putative, expressed	Os04g32880	11	3	232
	Carbon catabolite derepressing protein kinase, putative, expressed	Os03g17980	8	2	126
	SNF1-related protein kinase regulatory subunit beta-1, putative, expressed	Os03g20340	14	3	239
	Protochlorophyllide reductase B, chloroplast precursor, putative, expressed	Os10g35370	13	2	110
**12**	**SNF1-related protein kinase catalytic alpha subunit KIN10, putative, expressed**	Os05g45420	33	12	853
	protein kinase AKINbetagamma-2, putative, expressed	Os03g63940	19	5	716
	SNF1-related protein kinase regulatory subunit beta-1, putative, expressed	Os03g20340	18	4	275
	SNF1-related protein kinase regulatory subunit beta-1, putative, expressed	Os05g41220	19	4	261
	CBS domain containing protein, expressed	Os04g32880	7	3	160
**13**	**Mitogen-activated protein kinase homolog NTF3**	Os02g05480	22	10	751
	Cysteine protease 1 precursor, putative, expressed	Os04g57440	6	2	178

### Phosphorylation sites of PKs

**Figure 1:**
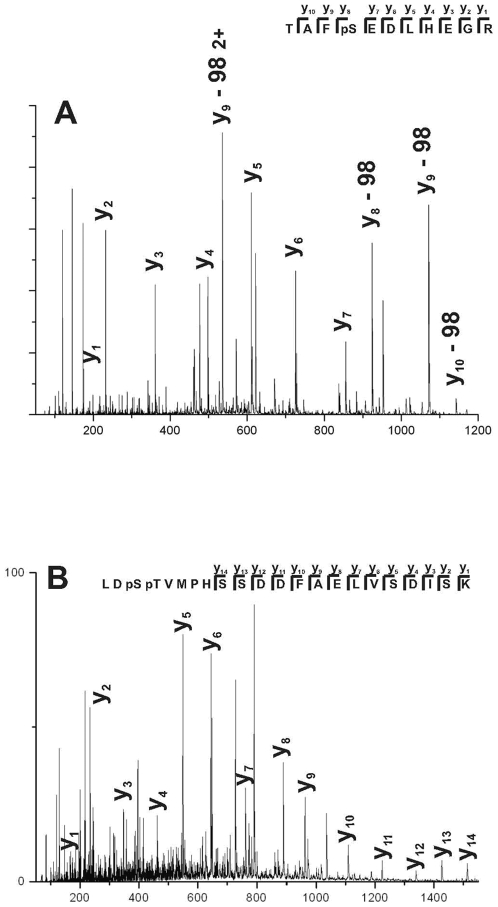
MS/MS spectra of rice phosphopeptides. (A) MS/MS spectrum produced from (M+2H)^2+^ of m/z 671.2 for phosphopeptides from rice Os01g14932. (B) MS/MS spectrum produced from (M+3H)^3+^ of m/z 906.1 for phosphopeptides from rice Os03g08550. Insert identifies the y-series ions for the peptides and are assigned in the spectra; neutral loss of H_3_PO_4_ (98 daltons) indicates the y fragment contains a phosphorylated amino acid.

Some of the TAP-tagged PKs showed evidence of phosphorylation in the MS/MS data. Many of these data were too weak, e.g. either peptide score was below than identity or peptide spectrum was not clear, to be conclusive but the peptides shown in Table 2 gave clear spectra and had the MOWSE scores above identity, examples of which are shown in Figure 1, indicating significant phosphorylation of these peptides. Generally the phosphopeptides are underrepresented in the generated complex peptide mixture during mass spectrometric studies [29] because of several reasons including selective suppression of their ionization/detection efficiencies in the presence of large amounts of unphosphorylated peptides and lower detection efficiencies of phosphopeptides as compared with their unphosphorylated cognates. The serine (pS) or threonine (pT) phosphorylation sites were also identified and are shown in Table 2. In PK Os03g08550 one of the phosphorylated peptide is located in the region between the transmembrane domain and the conserved PK domain. The second peptide is phosphorylated at adjacent serine and threonine positions (K.LD**pSpT**VMPFHSSDDFAELVSDISK.L) and is located within the protein kinase catalytic domain adjacent to the active site. Homology searches indicate that the amino acid sequence of the phosphorylated site is not conserved outside of the plant kingdom and the role of phosphorylation at this site is currently unknown. In both Os01g14932 and Os07g38810 (Table 2) the phosphorylated peptides are at the very C-terminus of the proteins. These phosphorylation sites are not in the conserved PK catalytic domains, are not homologous to non-plant PKs, and their biological significance is unknown at present. Since the phosphorylated peptides can show ion suppression, the lack of detection or inconclusive detection in other PKs is not proof that phosphorylated species are absent and can be checked by other means e.g radiolabeling. This is particularly problematic when a low percentage of the protein population is phosphorylated and therefore the phosphorylated peptide gives a weak MS/MS signal due to increased hydrophilicity and hence reduced retention of phosphopeptides on reversed-phase materials [29].

**Table 2:** Phosphorylated protein kinases

TAP tagged protein kinase	peptide	phosphorylation
**Os03g08550** (Suppl. table S3, no. 10); Leucine-rich repeat transmembrane protein	R.HK**pS**FDDDDLSNKPVLK.K	**pS**
	K.LD**pSpT**VMPFHSSDDFAELVSDISK.L	**pSpT**
**Os01g14932** (Suppl. table S3, no. 4); NAK-like ser/thr protein kinase	R.TAF**pS**EDLHEGR	**pS**
**Os07g38810** (Table 1, no. 3); Lectin protein kinase	NSISYISSASMGAISDI**pS**GGR	**pS**

The phosphorylated amino acid identified is shown in bold and underlined as a **pS** or **pT** when Ser or Thr are phosphorylated, respectively.

### Recurring and/or contaminant proteins

Background proteins from mock purifications from non-transgenic rice plants have been previously identified [24]. The contamination criterion of multiple recoveries of abundant proteins [30] was adopted in this and the earlier study [24] and used to expand the list of proteins that are found in more than 5% of the 129 cumulative purifications (Supplementary Table S2). These contaminating proteins include various ribosomal proteins, Rubisco, glyceraldehyde-3-phosphate dehydrogenase, glutamate decarboxylase, phosphoglycerate kinase and other proteins. However, these background proteins were recovered at fairly low amounts and generally did not significantly interfere with obtaining MS data from other proteins in the sample.

### Non-recovered or non-interacting PKs

Supplemental Table S1-C contains instances where no interacting proteins were found or instances where the TAP-tagged PKs was not recovered in sufficient amounts to be identified by MS/MS analysis but possible interacting proteins were found. In principle, the TAP-tag purification method requires that some TAP-tagged PK protein should be present to allow for the purification of a complex, so these interacting proteins could be contaminants, but are not among the recurring proteins. However, a lack of peptide identification from the TAP-tagged protein could be due to amounts too low to be identified in the MS/MS analysis because of various reasons including comparatively small amount of starting material and the nature and properties of the sample itself e.g. many hydrophobic proteins are less effectively digested by trypsin as compare to water soluble proteins. This leads to an occasional fortuitous identification of a peptide from an interacting protein. Again, these would require further verification.

## Discussion

### Individual protein complexes

#### SnRK1 catalytic α-subunit proteins

The evolutionarily conserved yeast SNF1 (Sucrose nonfermenting 1) and mammalian AMP-activated protein kinase (AMPK) proteins occur as heterotrimer complexes and are involved in regulating carbohydrate and lipid metabolism. Their plant orthologs also affect plant glucose, stress signaling, and development in plants [31]. The heterotrimer complex consists of the catalytic α-subunit (SNF1/AMPK/SnRK1), the targeting/adapter β-subunit (SIP1/SIP2/GAL83/AMPKβ), and regulatory γ subunit (SNF4/AMPKγ). Rice has three closely-related Snf1 Related Kinase (SnRK1) catalytic α-subunit genes (Os05g45420, Os08g37800, and Os03g17980) that appear to be orthologs of the Arabidopsis *Kin10* and *Kin11* genes. The β and γ subunits can occur as gene fusions in plants [32] or the β subunit can occur as a separate protein that interacts with the catalytic α-subunit in transient assays [33]. The rice genome has two β/γ fusion genes, four probable β-only subunits, and no obvious γ-only subunits.

Two separately TAP-tagged rice SnRK1 catalytic α-subunit proteins Os08g37800 (Table 1, no. 11) and Os05g45420 (Table 1, no. 12) were both found to interact with the two β/γ fusion subunits (Os03g63940 and Os04g32880) to form the expected αβ/γ heterotrimers. SnRK1 catalytic α-subunit protein Os08g37800 was predominantly associated with β/γ fusion subunit Os03g63940 while SnRK1 catalytic α-subunit protein Os05g45420 appeared to have more equal amounts of the two Os03g63940 and Os04g32880 β/γ fusion subunits in the protein population. As noted above, these are the only β/γ fusion proteins apparent in the rice genome [18]. Additionally, the initial purification for each tagged protein kinase found a single peptide for different members of the targeting/adapter β-subunit family in the two different complexes. SnRK1 α-subunit Os05g45420 was associated with β-subunit Os09g20010 (Supplementary Table S1-A, no. 12) while SnRK1 α-subunit Os08g37800 was associated with β-subunit Os05g41220 (Supplementary Table S1-A, no. 11). A second purification of each complex found multiple peptides for β-subunit Os03g20340 which were associated with both SnRK1 α-subunit Os08g37800 (Table 1, no. 11) and SnRK1 α-subunit Os05g45420 (Table 1, no. 12). Multiple peptides for β-subunit Os05g41220 were also associated with SnRK1 α-subunit Os05g45420 (Table 1, no. 12). No γ-only subunits were detected during this investigation.

Os03g17980, from the three gene family of SnRK1 catalytic α-subunit proteins, was also associated with SnRK1 catalytic α-subunit genes Os08g37800 (Table 1, no. 11). This finding, together with the finding of both β/γ and β subunits in the same protein complex indicates heterotrimers are associating *in vivo*. Assuming that the heterotrimer is the main form of the SnRK1 complex, the complexes containing the non-fused β-subunits are likely to interact with as yet unknown γ-subunits although none are recognizable in the genome or peptide sequence. Presumably these are less stably bound to the complex and were not recovered in our isolations. However, ββ interactions have been reported [34], raising the possibility of a lower abundance of αββ/γ complex.

### Receptor-Like Cytoplasmic Kinase VII

Rice Os07g49470 (Table 1, no. 6) is a member of the receptor-like cytoplasmic kinase VII subfamily (RLCK VII). It is closely related to several Arabidopsis genes, including the PBS1 gene (At5g13160) that is involved in defense signaling when the bacterial pathogen *Pseudomonas syringae* avirulence avrPphB protein is present [35]. The protein complex isolated with the TAP-tagged Os07g49470 protein includes two closely-related members of the karyopherin β (Kapβ, also known as importin β) protein family [Os07g38760 and Os03g49420 (Table 1, no. 6)]. Kapβ proteins are involved in the import and export of proteins and RNAs from the nucleus, often as a heterodimers with karyopherin α, which we did not detect in the isolated complex. They may also have roles in regulating the nuclear pore complex and nuclear envelope, mitosis and replication [36]. Kapαβ heterodimers work with Ran GTPase in the nuclear import or export of proteins with nuclear localization signal (NLS) or nuclear export signal (NES). The RLCK VII protein kinase Os07g49470 might be involved in regulating Kapβ activity or in phosphorylating proteins that interact with this protein complex. The absence of karyopherin α or Ran in the isolated protein complex may be due to less stable interactions or because the RLCK VII/Kapβ complex is involved in a process not requiring these proteins.

### PK1-related Protein Kinases

Rice PK1A-related Os05g02020 (Table 1, no. 7) and PK1B-related Os03g06330 (Table 1, no. 5) are members of a multi-gene family of PK1-related protein kinases that do not have well-defined biological roles as yet in rice or in Arabidopsis. Early reports indicated the Arabidopsis founding member PK1A was capable of both Ser/Thr and Tyr phosphorylation in bacterial extracts [37]. Gene annotation indicates either chloroplast localization or N-terminal protein myristoylation is possible. PK1A-related Os05g02020 and PK1B-related Os03g06330 were found to interact with DEAD/DEAH box helicase family protein Os03g61220 (Table 1, nos. 5 and 7). Rice PK1A-related Os05g02020 protein was found to interact with linker histone H1 and H5 protein Os03g58470; and another DEAD/DEAH box helicase Os01g43120 (Table 1, no. 7).

**Figure 2:**
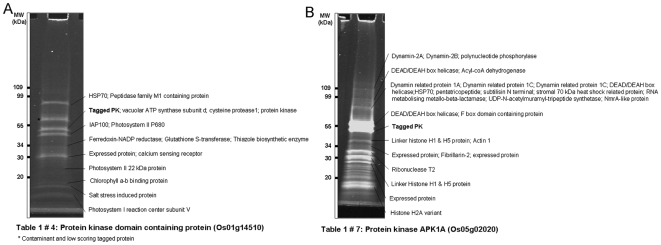
Sypro ruby stained TAP-purified protein complexes after SDS-PAGE. (A) TAP-purified rice Os01g14932 in-vivo protein complex from transgenic rice plants. (B) TAP-purified rice Os03g08550 in-vivo protein complex. The names of the proteins identified by mass spectrometry (MS) analysis of the digested peptides are shown in the bands. Standard protein molecular marker is shown on left side of the gel.

Additionally, PK1A-related Os05g02020 protein was found to interact with several members of the dynamin protein family [Dynamin-2A Os06g13820; dynamin-2B Os02g50550; dynamin-related protein 1A Os05g48240; dynamin-related protein 1C Os03g50520; and dynamin-related protein 1C Os10g41820 (Table 1, no. 7)]. Dynamin is a large GTPase protein involved in cell and organellar membrane budding, transport and fission [38]. This suggests that the PK1A-related Os05g02020 protein might be involved in regulating cellular membrane processes. Additional interacting proteins found include the RNA-related Ribonuclease T2 family protein Os09g36700 (Table 1, no. 7) (Figure 2B).

### Calcium-Dependent Protein Kinase

Rice Calcium-dependent protein kinase (CDPK) Os03g03660 TAP-tagged protein (Table 1, no. 1) was associated with nascent polypeptide-associated complex alpha subunit-like protein Os01g71230 (Table 1, no. 1) that has a possible ubiquitin-associated domain and this class of proteins is involved in many cellular processes [39].

### Leucine Rich Repeat (LRR) Protein Kinase

Rice Os01g14510 (Table 1, no. 4) is a LRR type kinase that has a transmembrane region very close to its N-terminus. It was found to interact with a CDPK Os03g03660 and a unique salt stress induced protein Os01g24710 (Figure 2A), suggesting a possible role in sensing salt stress but any functional role remains to be understood.

### Analysis of current and previous purifications of TAP-tagged PK complexes

Table 3 shows the summary of the percentages of successfully purified TAP-tagged protein kinases and of the associated protein complexes in the present investigation. Forty-five of the eighty-eight TAP-tagged protein kinases were recovered in amounts sufficient to identify the TAP-tagged PK by MS/MS analysis. Thirteen of the 45 PKs recovered contained biologically significant interacting proteins after subtracting the common contaminating proteins.

**Table 3:** Summary of Purification and Interaction Percentages of TAP-tagged Protein Kinases.

	Number	Percent
**88 PKs (This work)**		
Recovered TAP-tagged PKs (# peptides ≥2)	45/88	51
Identified Complexes (# peptides ≥2)	13/45	29
**41 PKs (Prior work** [**24**] **)**		
Recovered TAP-tagged PKs (# peptides ≥2)	38/41	93
Identified Complexes (# peptides ≥2)	16/38	42
**88 PKs combined with 41 previous PKs**		
Recovered TAP-tagged PKs (# peptides ≥2)	83/129	64
Identified Complexes (# peptides ≥2)	29/83	35

Our combined analysis of 129 TAP-tagged PKs, from the current 88 and previous 41 [24], indicates that the TAP method in transgenic rice plants recovers the TAP-tagged protein in amounts sufficient for MS/MS identification 64 percent of the time using the criteria that at least two peptides of each protein are identified. The 36 percent not recovered were predominantly due to lower levels of the TAP-tagged protein in the transgenic plants as opposed to purifications that failed for other reasons. A reduction to a 51% recovery rate of the TAP-tagged proteins in the current work from the prior recovery rate of 93% [24] is likely to be due to reduced amounts of the protein kinases in transgenic plants. This could be due in part to the prior selection of leaf expressed cDNAs available in the databases that tend to represent more abundant mRNAs due to their higher representation in cDNA libraries. Subsequent gene selections were based on microarray hybridization scores, proceeding from most abundant to least abundant. Reductions in the amount of protein purified affects the quality of the MS/MS identification of the protein as the number of peptides identified and MASCOT scores tend to correlate with the abundance of that protein in the purified sample. Low abundance recoveries often result in accurate single peptide identifications when using a fairly stringent MASCOT score requirement e.g. Supplementary Table S1-C shows four instances where the TAP-tagged PKs were identified only by one peptide with a high MASCOT score. However, the low level of protein that provides only a single identified peptide makes a distinction between a valid interaction and a contaminating protein more difficult. We provide data on single peptide identifications in the supplemental materials and at a supporting website (http://rkd.ucdavis.edu/) as we believe the data are useful but recognize that additional data is needed for confirmation of those interactions.

Of the 83 TAP-tagged proteins recovered from the 129 analyzed in our combined study, 29 were considered to have interacting proteins that had at least two peptides identified at high MASCOT scores. This amounts to 23 percent of the total 129 TAP-tagged proteins or 35% of the recovered TAP-tagged proteins. This sample size is large enough to be indicative of the frequency of success of the TAP-tagging method applied to protein kinases in plants, which are generally low abundance proteins, as inferred by their mRNA intensity scores from microarray analysis.

### Comparison to yeast two hybrid screening

Ninety three of the 129 RLePKs cDNAs used in this and an earlier study [24] have also been screened in a yeast two hybrid system against a library of rice cDNA prey vectors [27]. A comparison of these data sets found only 4 of the RLePK (bait or TAP-tagged) proteins interacted with one or more of the same proteins (TAP-tag complex or prey) in both techniques. Two of these are from the evolutionarily conserved casein kinase II and SNF1 protein complexes and thus have independent support for their interactions. The third protein (Os02g33450) appears in both data sets more than once making any conclusions more difficult about whether these are valid interactions or the result of a “sticky” protein in both systems.

The fourth interacting protein is unique in both data sets and identifies a new protein unique to rice that interacts with either RLePK protein Os03g01850 (found in the yeast two hybrid screen [27]) or Os03g02680 (found in our earlier TAP-tag report [24]). These two protein kinases are 97 percent identical and are considered to have effectively the same interactions (the identical kinases have not been screened in both systems). The amino acid sequence of this interacting Os07g12780 protein is annotated as a hypothetical rice protein and does not have close homology to proteins from any other species in the current databases including the fully sequenced *Arabidopsis* genome, suggesting the gene has evolved at least since the divergence of monocot and dicot plants. The interaction of the Os07g12780 protein is with the well-conserved cell division cycle control protein 2 (CDC2) TAP-tagged complex [24] that affects cell cycle [40]. Protein Os07g12780 appears to be an evolutionary addition to the function of the CDC2 protein complex in rice that will require further studies to define what its new role is.

As a percentage of the interacting proteins found in the TAP-tagged or yeast two hybrid systems, the same interacting proteins found by both methods were about 3 percent of the total interacting proteins. This percentage is similar to that found in a comparison of protein interaction methods for yeast protein interactions that found only 2,400 interactions were in common from a total of 80,000 interactions found from a variety of methods in yeast [41].

### Conclusions

Our studies demonstrate the applicability and limitations of the TAP-tag method in studying *in-planta* protein-protein interactions. Our data suggests although the TAP-tag methodology can be successful in plants [23], [24], [25], [26], there are concerns when small amounts of the TAP-tagged protein complexes recovered. The recovery of a single peptide from a protein, despite a high quality MASCOT score that reliably identifies the peptide, raises concerns about distinguishing interacting proteins from contaminants. More abundant amounts of a protein tend to result in the identification of multiple peptides in the MS/MS analysis. Requiring at least two peptides of a protein to be identified as a valid interaction reduces the chances of recovering a single peptide from a low level contaminant. Single peptide identifications can be from valid interactions and are therefore still useful in a database, but are included only in the supplemental tables and would require further experimental verification of the possible interaction.

The question of why such low levels of protein and protein complexes are often recovered still remains. There is some concern about the functionality of the fusion proteins. A report of a genetic complementation analysis of seven TAP-tagged proteins found only two of the fusion proteins fully complemented their corresponding mutation in *Arabidopsis* plants [25]. Three additional fusions partially complemented their mutation but this latter result is difficult to interpret as the percentage of functional activity needed for partial complementation is unclear. This suggests the TAP-tag fusions may interfere with the ability to form functional proteins or protein complexes. A second concern is the relatively lengthy purification process in the TAP-tag method, as the time required to finish the purification averages about 5 hrs in our experience. Presumably only very stable protein complexes survive this lengthy procedure. Given the fairly high frequency of recovery of the TAP-tagged protein, the ability of the fusion protein to form an abundant and stable protein complex appears to be the most important variable affecting the recovery of a protein complex by this method. Additional factors that might affect the efficiency of protein complex formation have been discussed previously [24]. Our results might be specific to the protein kinase family we have investigated but suggest the need for improved methods for the more rapid recovery of low abundance target proteins and their interacting proteins.

Comparisons of protein interaction methods for protein interactions in yeast found only 2,400 interactions were in common from a total of 80,000 interactions found from a variety of methods in yeast [41]. The authors conclude that multiple protein-interaction methods are needed and that confirmation by more than one method provides the most reliable data [41]. Larger protein interaction data sets are needed in plants to increase the probability of multiple confirmations.

## Materials and Methods

The protein kinases in the rice genome were previously annotated using release 3 of the rice genome annotation (http://www.tigr.org/tdb/e2k1/osa1/) and full length cDNA data [42]. This set consists of 1,429 unique protein kinases (http://rkd.ucdavis.edu/) with an additional 81 forms due to alternative splicing. Eighty eight of the PKs cDNAs were chosen as a representative set of protein kinases to determine the protein interactions in signaling pathways in leaves. These PK cDNAs were fused in-frame with a N-terminal TAP-tag for constitutive expression from the maize ubiquitin promoter in transgenic rice plants and purified using the TAP purification methods previously reported [24]. In the case of receptor like kinases (RLKs) with transmembrane regions, only the intracellular domains of the RLKs were cloned to avoid the difficulties of purifying membrane-bound protein complexes [20]. Plants were grown in the greenhouse with natural and supplemental (sodium and metal halide lamps) lights on a 16 h day at a light intensity of 500–1000 µmol PAR per m^2^ s^−1^ at a day/night temperature of 30°C/24°C.

### Tandem affinity purification and separation of protein mixtures by SDS-PAGE

The TAP-tagged kinases were purified from 6 to 8 weeks old T1 plants using a previously published TAP procedure for plants [24]. The purified samples were resolved by SDS-PAGE, stained with Sypro Ruby fluorescent dye and visualized by UV. Two independent purifications were performed for ten of the TAP-tagged kinases showing interesting protein interactions (Table 1) to check the reproducibility of the method.

### In-gel digestion, Mass Spectrometry and MS data analysis

The Sypro Ruby stained bands from the SDS-PAGE gel were excised and the trypsin-digested peptides were subjected to LC/MS-MS as described [23], [24], [43]. Briefly, gel pieces were digested by trypsin (no. V5111, Promega, Madison, WI) and the digested peptides were extracted in 5% formic acid/50% acetonitrile and separated using C18 reversed phase LC column (Dionex, Sunnyvale, CA). A Q-TOF Ultima tandem mass spectrometer (Waters) with electrospray ionization was used to analyze the eluting peptides. The system was user controlled employing MassLynx software (v 4.0, Waters) in data-dependant acquisition mode with the following parameters: 1-sec survey scan (380–1900 Da) followed by up to three 2.4-sec MS/MS acquisitions (60–1900 Da). The instrument was operated at a mass resolution of 8000. The instrument was calibrated using the fragment ion masses of doubly protonated Glu-fibrinopeptide.

The peak lists of MS/MS data were generated using Distiller (Matrix Science, v1.9.0, London, UK) using charge state recognition and deisotoping with the other default parameters for Q-TOF data. Data base searches of the acquired MS/MS spectra were performed using Mascot (Matrix Science, v1.9.0, London, UK). A second database, MSDB (Mass Spectrometry protein sequence Data Base) was also utilized. When using the MSDB database (a comprehensive, non-identical protein sequence database maintained by the Proteomics Department at the Hammersmith Campus of Imperial College London which combines entries from TREMPL, SWISSPOT and GENBANK) the taxonomy filter was set as Vindiplantae (green plants) for the taxonomic category and searched 2081917 sequences or 677709849 residues (MSDB 20051114). The rice genome sequences (http://www.tigr.org/tbe/e2k1/osa1/) were also utilized. Search parameters used were: no restrictions on protein molecular weight or pI, enzymatic specificity was set to trypsin, and methionine oxidation and phosphorylation were allowed as variable peptide modifications. Mass accuracy settings were 0.15 daltons for peptide mass and 0.12 daltons for fragment ion masses. MASCOT peptide scores equal to or higher than the “identity” MASCOT score (typically 39 to 41 during this investigation) for that peptide were required for a peptide to be considered a valid identification of a protein. The MOWSE score was checked manually for every peptide and only MOWSE scores above the identity threshold were considered valid [28]. For single-peptide-based protein identifications or post-translationally modified peptides, the sequence identified and the precursor m/z value observed along with the score is provided in Supplementary Table S3. Mass Spectra for the identified proteins are also provided (Supplementary Table S4). Each unique peptide was considered as only one peptide even if it was recovered multiple times in a sample. The interaction data and additional information about rice protein kinases is available on the project website (rkd.ucdavis.edu). All of the MS/MS spectra of peptides that were assigned by MASCOT as potentially having been phosphorylated were examined manually to verify the assignment. The concurrent loss of H_3_PO_4_ (98 daltons) along with the expected peptide backbone fragmentation was used to validate the MASCOT assignment.

## Supporting Information

Table S1 A. TAP-tagged protein kinases and interacting proteins from Table I when single peptide identifications are included B. TAP-tagged protein kinase purifications where only single peptides for a potential interacting protein or peptides from a unique isoform member of a family of abundant proteins were recovered C. TAP-tagged protein kinase purifications where either the tagged protein or associated protein(s) were not detected
(0.84 MB DOC)

Table S2 Proteins subtracted from the protein-protein interaction data base as recurring/contaminant proteins
(0.06 MB DOC)

Table S3 Sequence identified, precursor m/z values and scores for single-peptide-based identified proteins
(0.07 MB XLS)

Table S4 Mass-spectra for the single-peptide-based identified proteins
(1.09 MB XLS)

File S1 Gel pictures of purifications that identified protein complexes.
(0.11 MB PDF)
