# Supplementary material for: Protein-Protein Interactions of Tandem Affinity Purified Protein Kinases from Rice
Source: PLoS One. 2009 Aug 19;4(8):e6685. doi: 10.1371/journal.pone.0006685 (PMC2723914; doi:10.1371/journal.pone.0006685)
Supplement: Table S1 — A. TAP-tagged protein kinases and interacting proteins from Table I when single peptide identifications are included B. TAP-tagged protein kinase purifications where only single peptides for a potential interacting protein or peptides from a unique isoform member of a family of abundant proteins were recovered C. TAP-tagged protein kinase purifications where either the tagged protein or associated protein(s) were not detected (0.84 MB DOC) [file pone.0006685.s001.doc]

**A. TAP-tagged protein kinases and interacting proteins from Table I when single peptide identifications are included**

| S. No. | Protein Name | TIGR_id | SwissProt_id | No. of peptides | Score | % coverage |
| --- | --- | --- | --- | --- | --- | --- |
| **1** | **Calcium-dependent protein kinase, isoform 11, putative, expressed** | **Os03g03660** | Q8H9A7_ORYSA | 11 | 789 | 20 |
|  | Calcium-dependent protein kinase, isoform AK1, putative | Os03g57510 | Q852N6_ORYSA | 2 | 152 | 3 |
|  | DNA-binding protein, 42kDa containing protein, expressed | Os05g28280 | Q5W6H1_ORYSA | 1 | 73 | 2 |
|  | Catalase-1, putative, expressed | Os03g03910 | Q8LSP8_ORYSA | 1 | 63 | 2 |
|  | Serine hydroxymethyltransferase, mitochondrial precursor, putative, expressed | Os03g52840 | Q7Y1F0_ORYSA | 6 | 450 | 21 |
|  | Aldehyde dehydrogenase, mitochondrial precursor, putative, expressed | Os06g15990 | Q9FRX7_ORYSA | 2 | 141 | 5 |
|  | Purine-rich single-stranded DNA-binding protein, putative, expressed | Os01g15600 | Q5NAR8_ORYSA | 1 | 49 | 3 |
|  | Fructose-bisphosphate aldolase, chloroplast precursor, putative, expressed | Os11g07020 | AAX95072 | 3 | 225 | 8 |
|  | Malate dehydrogenase, glyoxysomal precursor, putative, expressed | Os03g56280 | Q7XZW5_ORYSA | 3 | 95 | 14 |
|  | Nascent polypeptide-associated complex alpha subunit-like protein 3, putative, expressed | Os01g71230 | Q8RUI4_ORYSA | 2 | 194 | 18 |
|  | ATP-dependent Clp protease proteolytic subunit, putative, expressed | Os05g51450 | Q6L4S1_ORYSA | 1 | 83 | 3 |
|  | 2-cys peroxiredoxin BAS1, chloroplast precursor, putative, expressed | Os02g33450 | Q6ER94_ORYSA | 1 | 51 | 5 |
|  | CBS domain containing protein, expressed | Os09g02710 | Q6YYV0_ORYSA | 1 | 79 | 6 |
|  | Initiation factor 2 subunit family protein, expressed | Os10g25320 | Q7XEY8_ORYSA | 1 | 47 | 3 |
|  | heavy metal-associated domain containing protein, expressed | Os05g27100 | Q5WMQ0_ORYSA | 1 | 109 | 4 |
|  | 6,7-dimethyl-8-ribityllumazine synthase family protein, expressed | Os04g42000 | Q7XUK6_ORYSA | 1 | 41 | 9 |
|  | ATP-dependent Clp protease proteolytic subunit 2, putative, expressed | Os06g04530 | Q5VQ13_ORYSA | 1 | 83 | 5 |
|  | Ycf52, putative, expressed | Os05g40260 | Q5KQI6_ORYSA | 1 | 77 | 4 |
| **2** | **Lectin receptor kinase 7, putative, expressed** | **Os07g38800** | Q8LIP2_ORYSA | 10 | 889 | 26 |
|  | Heat shock cognate 70 kDa protein, putative, expressed | Os11g47760 | S53126 | 10 | 1273 | 29 |
|  | RuBisCO subunit binding-protein beta subunit, chloroplast precursor, putative, expressed | Os06g02380 | Q9LWT6_ORYSA | 16 | 2521 | 58 |
|  | RuBisCO subunit binding-protein alpha subunit, chloroplast precursor, putative, expressed | Os03g64210 | Q7X9A7_ORYSA | 11 | 1407 | 36 |
|  | Chaperonin CPN60-1, mitochondrial precursor, putative, expressed | Os10g32550 | Q8H903_ORYSA | 13 | 1038 | 36 |
|  | Chaperonin CPN60-2, mitochondrial precursor, putative, expressed | Os05g46290 | Q5TKQ5_ORYSA | 4 | 188 | 7 |
|  | ABC transporter family protein, putative, expressed | Os01g74470 | Q8GU74_ORYSA | 1 | 46 | <1 |
|  | 26S proteasome non-ATPase regulatory subunit 3, putative, expressed | Os08g43640 | Q6ZJI3_ORYSA | 2 | 96 | 5 |
|  | 26S protease regulatory subunit 6A homolog, putative, expressed | Os06g07630 | Q5SNC0_ORYSA | 1 | 65 | 3 |
|  | Actin-7, putative, expressed | Os11g06390 | Q67G20_ORYSA | 5 | 347 | 19 |
|  | IAP100, putative, expressed | Os10g35030 | Q9FWV4_ORYSA | 2 | 178 | 8 |
|  | Glutamate-1-semialdehyde 2,1-aminomutase, chloroplast precursor, putative, expressed | Os08g41990 | Q6YZE2_ORYSA | 2 | 146 | 6 |
|  | Mov34/MPN/PAD-1 family protein, expressed | Os04g56654 | Q8W424_ORYSA | 1 | 107 | 5 |
|  | Protein kinase domain containing protein, expressed | Os10g37190 | Q7XCT8_ORYSA | 1 | 49 | 3 |
|  | Ferredoxin-NADP reductase, leaf isozyme, chloroplast precursor, putative, expressed | Os02g01340 | Q6ZFJ3_ORYSA | 2 | 190 | 9 |
|  | Chlorophyll A-B binding protein, expressed | Os11g13890 | AAX95978 | 2 | 90 | 9 |
|  | Chlorophyll a-b binding protein 8, chloroplast precursor, putative, expressed | Os02g10390 | Q6H746_ORYSA | 1 | 74 | 9 |
|  | expressed protein | Os05g33280 | Q6ATY4_ORYSA | 1 | 79 | 5 |
|  | Actin-1, putative, expressed | Os03g50890 | ATRZ1 | 1 | 58 | 4 |
|  | expressed protein | Os07g37240 | T02877 | 1 | 66 | 6 |
|  | Photosystem II 22 kDa protein, chloroplast precursor, putative, expressed | Os01g64960 | Q943K1_ORYSA | 1 | 55 | 5 |
|  | fiber protein Fb15, putative, expressed | Os02g07910 | Q6EUQ5_ORYSA | 1 | 61 | 7 |
| **3** | **Lectin protein kinase, putative, expressed** | **Os07g38810** | Q7F226_ORYSA | 9 | 1158 | 26 |
|  | RuBisCO subunit binding-protein beta subunit, chloroplast precursor, putative, expressed | Os06g02380 | Q9LWT6_ORYSA | 8 | 950 | 25 |
|  | RuBisCO subunit binding-protein alpha subunit, chloroplast precursor, putative, expressed | Os03g64210 | Q7X9A7_ORYSA | 4 | 398 | 9 |
|  | Chaperonin CPN60-1, mitochondrial precursor, putative, expressed | Os10g32550 | Q8H903_ORYSA | 2 | 102 | 5 |
|  | Cysteine protease 1 precursor, putative, expressed | Os04g57440 | Q7XMQ3_ORYSA | 2 | 157 | 6 |
|  | SHR5-receptor-like kinase, putative, expressed | Os05g16740 | Q6F2P3_ORYSA | 1 | 46 | 1 |
| **4** | **Protein kinase domain containing protein** | **Os01g14510** | Q9FTL1_ORYSA | 5 | 375 | 22 |
|  | Heat shock cognate 70 kDa protein, putative, expressed | Os11g47760 | S53126 | 10 | 1007 | 27 |
|  | Peptidase family M1 containing protein, expressed | Os03g60460 | Q84TA3_ORYSA | 1 | 102 | 3 |
|  | Vacuolar ATP synthase subunit d, putative, expressed | Os01g40470 | Q8RU33_ORYSA | 1 | 99 | 4 |
|  | Cysteine protease 1 precursor, putative, expressed | Os04g57440 | KHRZOA | 1 | 81 | 2 |
|  | Protein kinase domain containing protein, expressed | Os05g25390 | Q5W6V9_ORYSA | 1 | 46 | 2 |
|  | IAP100, putative, expressed | Os10g35030 | Q9FWV4_ORYSA | 2 | 159 | 8 |
|  | Photosystem II P680 chlorophyll A apoprotein, putative, expressed | Os08g15260 | Q6K455_ORYSA | 1 | 69 | 2 |
|  | Ferredoxin-NADP reductase, leaf isozyme, chloroplast precursor, putative, expressed | Os06g01850 | T04349 | 2 | 192 | 9 |
|  | Glutathione S-transferase, N-terminal domain containing protein, expressed | Os08g44400 | Q6YZJ0_ORYSA | 1 | 75 | 3 |
|  | Thiazole biosynthetic enzyme 1-1, chloroplast precursor, putative, expressed | Os07g34570 | Q7XHY5_ORYSA | 1 | 68 | 4 |
|  | expressed protein | Os07g37240 | T02877 | 1 | 100 | 6 |
|  | calcium sensing receptor, putative, expressed | Os02g49680 | Q6S3G1_ORYSA | 1 | 39 | 3 |
|  | Photosystem II 22 kDa protein, chloroplast precursor, putative, expressed | Os01g64960 | Q943K1_ORYSA | 1 | 90 | 5 |
|  | Chlorophyll a-b binding protein CP24 10B, chloroplast precursor, putative, expressed | Os04g38410 | Q7XV11_ORYSA | 1 | 64 | 4 |
|  | Salt stress-induced protein, putative, expressed | Os01g24710 | SALT_ORYSA | 2 | 120 | 18 |
|  | Photosystem I reaction center subunit V, chloroplast precursor, putative, expressed | Os09g30340 | Q69QR6_ORYSA | 1 | 41 | 6 |
| **5** | **Protein kinase APK1B, chloroplast precursor, putative, expressed** | **Os03g06330** | Q8H8B3_ORYSA | 11 | 1560 | 70 |
|  | D-mannose binding lectin family protein, expressed | Os04g53994 | Q5JQW1_ORYSA | 2 | 67 | <1 |
|  | Protein kinase APK1B, chloroplast precursor, putative, expressed | Os06g48980 | Q5Z823_ORYSA | 1 | 99 | 5 |
|  | Protein kinase APK1A, chloroplast precursor, putative, expressed | Os01g40590 | Q8S1E8_ORYSA | 2 | 95 | 3 |
|  | Linker histone H1 and H5 family protein, expressed | Os03g58470 | Q851P9_ORYSA | 1 | 77 | 3 |
|  | Histone H2A variant, putative, expressed | Os10g28230 | Q7XEK5_ORYSA | 1 | 52 | 7 |
|  | hypothetical protein | Os03g47090 | Q5VPN2_ORYSA | 1 | 47 | 4 |
|  | Potassium channel AKT2/3, putative, expressed | Os05g35410 | Q5TKJ4_ORYSA | 1 | 47 | 1 |
|  | 28 kDa ribonucleoprotein, chloroplast, putative, expressed | Os09g39180 | Q650W6_ORYSA | 1 | 41 | 3 |
|  | Heat shock cognate 70 kDa protein, putative, expressed | Os11g47760 | Q40693_ORYSA | 8 | 747 | 20 |
|  | DEAD/DEAH box helicase family protein, expressed | Os03g61220 | Q8SAX7_ORYSA | 2 | 151 | 3 |
|  | tRNA synthetase class I family protein, putative, expressed | Os10g32570 | Q8H905_ORYSA | 1 | 40 | 2 |
| **6** | **Serine/threonine-protein kinase RLCKVII** | **Os07g49470** | Q6Z3X8_ORYSA | 6 | 594 | 30 |
|  | HEAT repeat family protein, karyopherin-beta 3 variant expressed | Os07g38760 | Q6ZL37_ORYSA | 4 | 336 | 6 |
|  | HEAT repeat family protein, expressed | Os03g49420 | AAX95644 | 4 | 220 | 6 |
|  | expressed protein | Os01g68720 | Q8RZX3_ORYSA | 1 | 49 | 8 |
|  | NB-ARC domain containing protein | Os11g24170 | AAX96627 | 1 | 42 | <1 |
| 7 | **Protein kinase APK1A, chloroplast precursor** | **Os05g02020** | Q65XV8_ORYSA | 10 | 2016 | 66 |
|  | Dynamin-2A, putative, expressed | Os06g13820 | Q654U5_ORYSA | 14 | 1210 | 24 |
|  | Dynamin-2B, putative, expressed | Os02g50550 | Q6Z5P1_ORYSA | 13 | 816 | 18 |
|  | polynucleotide phosphorylase, putative, expressed | Os07g07310 | Q69LE7_ORYSA | 1 | 76 | 2 |
|  | DEAD/DEAH box helicase family protein, expressed | Os03g61220 | Q8SAX7_ORYSA | 8 | 1056 | 23 |
|  | Acyl-CoA dehydrogenase, C-terminal domain containing protein, expressed | Os07g47820 | Q6ZDX3_ORYSA | 1 | 52 | 1 |
|  | Dynamin-related protein 1A, putative, expressed | Os05g48240 | Q6I614_ORYSA | 7 | 819 | 19 |
|  | Dynamin-related protein 1C, putative, expressed | Os03g50520 | Q8W315_ORYSA | 7 | 584 | 22 |
|  | Dynamin-related protein 1C, putative, expressed | Os10g41820 | Q7XBZ9_ORYSA | 8 | 498 | 18 |
|  | DEAD/DEAH box helicase family protein, expressed | Os01g43120 | Q5ZBH5_ORYSA | 6 | 386 | 16 |
|  | Heat shock cognate 70 kDa protein, putative, expressed | Os11g47760 | S53126 | 4 | 280 | 11 |
|  | pentatricopeptide, putative, expressed | Os03g63910 | Q75IP8_ORYSA | 3 | 97 | 5 |
|  | Subtilisin N-terminal Region family protein, expressed | Os07g39020 | Q6ZL89_ORYSA | 1 | 83 | 1 |
|  | Stromal 70 kDa heat shock-related protein, chloroplast precursor, putative, expressed | Os12g14070 | Q84P99_ORYSA | 1 | 67 | 6 |
|  | RNA-metabolising metallo-beta-lactamase family protein, expressed | Os02g33610 | Q6ER78_ORYSA | 1 | 65 | 2 |
|  | UDP-N-acetylmuramyl-tripeptide synthetases family protein, expressed | Os10g40130 | Q94LU9_ORYSA | 1 | 57 | 6 |
|  | NmrA-like family protein, expressed | Os06g49120 | Q5Z8V8_ORYSA | 1 | 56 | 2 |
|  | DEAD/DEAH box helicase, putative, expressed | Os03g01830 | Q8H7P8_ORYSA | 1 | 77 | 2 |
|  | F-box domain containing protein | Os11g10370 | Q9LLP2_ORYSA | 1 | 60 | 2 |
|  | linker histone H1 and H5 family protein, expressed | Os03g58470 | Q851P9_ORYSA | 4 | 249 | 28 |
|  | Actin-1, putative, expressed | Os03g50890 | ATRZ1 | 1 | 49 | 4 |
|  | expressed protein | Os02g22070 | Q6Z867_ORYSA | 2 | 125 | 9 |
|  | Fibrillarin-2, putative, expressed | Os05g08360 | Q6AT27_ORYSA | 1 | 59 | 3 |
|  | expressed protein | Os01g68720 | Q8RZX3_ORYSA | 1 | 56 | 8 |
|  | Ribonuclease T2 family protein, expressed | Os09g36700 | Q69JF3_ORYSA | 1 | 119 | 7 |
|  | linker histone H1 and H5 family protein, expressed | Os04g18090 | Q7XM53_ORYSA | 1 | 45 | 4 |
|  | expressed protein | Os01g37210 | Q6Z338_ORYSA | 1 | 41 | 9 |
|  | Histone H2A variant, putative, expressed | Os10g28230 | Q7XEK5_ORYSA | 1 | 59 | 6 |
| **8** | **Protein kinase domain containing protein, expressed** | **Os06g50100** | Q5Z9Q3_ORYSA | 8 | 960 | 37 |
|  | Heat shock cognate 70 kDa protein, putative, expressed | Os01g62290 | Q943K7_ORYSA | 5 | 360 | 12 |
|  | Heat shock cognate 70 kDa protein, putative, expressed | Os11g47760 | S53126 | 5 | 340 | 11 |
|  | Protein kinase domain containing protein, expressed | Os07g43560 | Q7X8H4_ORYSA | 2 | 117 | 4 |
| **9** | **Protein kinase domain containing protein, expressed** | **Os01g67340** | Q5N854_ORYSA | 8 | 1167 | 51 |
|  | Heat shock protein 82, putative, expressed | Os08g39140 | Q5QLP0_ORYSA | 1 | 72 | 2 |
|  | D-alanine ligase family, putative, expressed | Os07g49110 | Q7XI67_ORYSA | 1 | 107 | 1 |
|  | Elongation factor 2, putative, expressed | Os02g32030 | Q6H4L2_ORYSA | 1 | 46 | 1 |
|  | Heat shock cognate 70 kDa protein, putative, expressed | Os11g47760 | Q40693_ORYSA | 5 | 375 | 14 |
|  | Transketolase, chloroplast precursor, putative, expressed | Os06g04270 | Q5VNW1_ORYSA | 2 | 156 | 5 |
|  | Vacuolar ATP synthase catalytic subunit A, putative, expressed | Os06g45120 | Q651T8_ORYSA | 1 | 70 | 1 |
|  | Aldehyde dehydrogenase, mitochondrial precursor, putative, expressed | Os06g15990 | Q9FRX7_ORYSA | 2 | 124 | 5 |
|  | Alanine aminotransferase 2, putative, expressed | Os07g01760 | Q69UU3_ORYSA | 1 | 72 | 2 |
|  | Serine hydroxymethyltransferase, mitochondrial precursor, putative, expressed | Os03g52840 | Q7Y1F0_ORYSA | 1 | 43 | 2 |
|  | Eukaryotic initiation factor 4A, putative, expressed | Os02g05330 | Q9AR32_ORYSA | 1 | 41 | 5 |
|  | IAP100, putative, expressed | Os10g35030 | Q9FWV4_ORYSA | 1 | 108 | 5 |
|  | oxidoreductase, zinc-binding dehydrogenase family protein, expressed | Os08g43190 | Q6ZBH2_ORYSA | 1 | 90 | 4 |
|  | Glutamine synthetase, chloroplast precursor, putative, expressed | Os04g56400 | AJRZQD | 1 | 85 | 3 |
|  | Glutamate-1-semialdehyde 2,1-aminomutase, chloroplast precursor, putative | Os08g41990 | Q6YZE2_ORYSA | 1 | 62 | 3 |
|  | Actin-1, putative, expressed | Os03g50890 | ATRZ1 | 1 | 105 | 4 |
|  | Malate dehydrogenase, glyoxysomal precursor, putative, expressed | Os03g56280 | Q7XZW5_ORYSA | 3 | 242 | 17 |
|  | mRNA-binding protein precursor, putative, expressed | Os07g11110 | Q8GTK8_ORYSA | 1 | 88 | 4 |
|  | expressed protein | Os08g44000 | Q6Z3F9_ORYSA | 1 | 66 | 4 |
|  | Triosephosphate isomerase, chloroplast precursor, putative, expressed | Os09g36450 | Q69K00_ORYSA | 2 | 145 | 14 |
|  | Ribonuclease T2 family protein, expressed | Os09g36680 | Q8RYA7_ORYSA | 1 | 97 | 5 |
|  | Carbonic anhydrase, chloroplast precursor, putative, expressed | Os01g45274 | T03254 | 1 | 68 | 4 |
|  | 2-cys peroxiredoxin BAS1, chloroplast precursor, putative, expressed | Os02g33450 | Q6ER94_ORYSA | 3 | 177 | 17 |
|  | Chlorophyll a-b binding protein 8, chloroplast precursor, putative, expressed | Os02g10390 | Q6H746_ORYSA | 1 | 93 | 9 |
|  | CBS domain containing protein, expressed | Os03g52690 | Q84R32_ORYSA | 2 | 117 | 14 |
| **10** | **WAK-like kinase, putative, expressed** | **Os03g12470** | Q8H7R6_ORYSA | 9 | 616 | 17 |
|  | Heat shock cognate 70 kDa protein, putative, expressed | Os11g47760 | S53126 | 16 | 1342 | 47 |
|  | Stromal 70 kDa heat shock-related protein, chloroplast precursor, putative, expressed | Os12g14070 | Q84P99_ORYSA | 1 | 71 | 6 |
|  | RuBisCO subunit binding-protein beta subunit, chloroplast precursor, putative, expressed | Os06g02380 | Q9LWT6_ORYSA | 5 | 579 | 15 |
|  | Chaperonin CPN60-1, mitochondrial precursor, putative, expressed | Os10g32550 | Q8H903_ORYSA | 6 | 338 | 16 |
|  | RuBisCO subunit binding-protein alpha subunit, chloroplast precursor, putative, expressed | Os03g64210 | Q7X9A7_ORYSA | 1 | 74 | 2 |
|  | Cysteine protease 1 precursor, putative, expressed | Os04g57440 | Q7XMQ3_ORYSA | 2 | 139 | 6 |
|  | hypothetical protein | Os10g21540 | Q8W2W3_ORYSA | 1 | 43 | 2 |
| **11** | **Carbon catabolite derepressing protein kinase, putative, expressed** | **Os08g37800** | Q852Q1_ORYSA | 13 | 1056 | 35 |
|  | Protein kinase AKINbetagamma-2, putative, expressed | Os03g63940 | Q84M80_ORYSA | 7 | 1070 | 32 |
|  | CBS domain containing protein, expressed | Os04g32880 | Q7XV93_ORYSA | 1 | 84 | 9 |
|  | Potassium channel AKT2/3, putative, expressed | Os05g35410 | Q5TKJ4_ORYSA | 1 | 51 | 1 |
|  | SNF1-related protein kinase regulatory beta subunit 1, putative, expressed | Os05g41220 | Q6F337_ORYSA | 1 | 70 | 16 |
| **12** | **SNF1-related protein kinase catalytic alpha subunit KIN10, putative, expressed** | **Os05g45420** | Q852Q2_ORYSA | 12 | 853 | 33 |
|  | protein kinase AKINbetagamma-2, putative, expressed | Os03g63940 | Q84M80_ORYSA | 5 | 716 | 19 |
|  | CBS domain containing protein, expressed | Os04g32880 | Q7XV93_ORYSA | 3 | 253 | 23 |
|  | ATP-dependent Clp protease proteolytic subunit, putative, expressed | Os05g51450 | Q6L4S1_ORYSA | 1 | 52 | 3 |
|  | SNF1-related protein kinase regulatory beta subunit 2, putative, expressed | Os09g20010 | Q69NJ8_ORYSA | 2 | 103 | 19 |
| **13** | **OsMPK14 - putative MAPK based on amino acid sequence homology, expressed** | **Os02g05480** | Q6Z437_ORYSA | 10 | 751 | 22 |
|  | RuBisCO subunit binding-protein beta subunit, chloroplast precursor, putative, expressed | Os06g02380 | Q9LWT6_ORYSA | 4 | 415 | 12 |
|  | Chaperonin CPN60-2, mitochondrial precursor, putative, expressed | Os05g46290 | Q5TKQ5_ORYSA | 1 | 64 | 1 |
|  | Cysteine protease 1 precursor, putative, expressed | Os04g57440 | Q7XMQ3_ORYSA | 2 | 178 | 6 |

**B. TAP-tagged protein kinase purifications where only single peptides for a potential interacting protein or peptides from a unique isoform member of a family of abundant proteins were recovered**

| S. No. | Protein Name | TIGR_id | SwissProt_id | No. of peptides | Score | % coverage |
| --- | --- | --- | --- | --- | --- | --- |
| **1** | **Serine/threonine-protein kinase SAPK3, putative, expressed** | **Os10g41490** | Q7XC29_ORYSA | 14 | 987 | 41 |
|  | retrotransposon protein, putative, Ty3-gypsy subclass | Os11g26460 | AAX95930 | 1 | 39 | <1 |
| **2** | **Protein kinase domain containing protein, expressed** | **Os05g32360** | Q6I5Y0_ORYSA | 4 | 334 | 13 |
|  | retrotransposon protein, putative, Ty3-gypsy subclass | Os10g09430 | Q6ZFS9_ORYSA | 1 | 44 | 5 |
|  | ATP-dependent Clp protease proteolytic subunit, putative, expressed | Os05g51450 | Q6L4S1_ORYSA | 1 | 75 | 3 |
| **3** | **Protein kinase family protein** | **Os01g56330** | Q94EF4_ORYSA | 17 | 1103 | 28 |
|  | Heat shock cognate 70 kD protein, putative, expressed | Os11g47760 | S53126 | 22 | 1431 | 45 |
|  | Stromal 70 kD heat shock-related protein, chloroplast precursor, putative, expressed | Os12g14070 | Q84P99_ORYSA | 2 | 100 | 8 |
|  | Chlorophyll a-b binding protein 8, chloroplast precursor, putative, expressed | Os02g10390 | Q6H746_ORYSA | 1 | 44 | 9 |
| **4** | **NAK-like ser/thr protein kinase, putative, expressed** | **Os01g14932** | Q5NBQ2_ORYSA | 16 | 1010 | 24 |
|  | Leucine Rich Repeat family protein | Os02g05940 | Q5UD37_ORYRU | 1 | 52 | 1 |
|  | strubbelig receptor family 5, putative, expressed | Os09g38700 | Q653R1_ORYSA | 1 | 52 | 4 |
|  | Leucine Rich Repeat family protein, expressed | Os02g05910 | Q5UD34_ORYRU | 1 | 51 | 1 |
|  | Protein kinase domain containing protein, expressed | Os07g35370 | Q84SG9_ORYSA | 1 | 45 | 2 |
|  | F-box domain containing protein | Os11g10370 | Q9LLP2_ORYSA | 1 | 52 | 2 |
|  | hypothetical protein | Os03g60660 | Q84TB1_ORYSA | 1 | 57 | 7 |
|  | transposon protein, putative, CACTA, En/Spm sub-class, expressed | Os01g09220 | Q5SNE7_ORYSA | 1 | 44 | 1 |
|  | Shugoshin C terminus family protein, expressed | Os02g55570 | Q69QY9_ORYSA | 1 | 44 | 1 |
| **5** | **Protein kinase domain containing protein, expressed** | **Os08g02996** | Q6YX21_ORYSA | 10 | 723 | 23 |
|  | Chaperonin CPN60-1, mitochondrial precursor, putative, expressed | Os10g32550 | Q8H903_ORYSA | 8 | 481 | 19 |
|  | hypothetical protein | Os10g21540 | Q8W2W3_ORYSA | 1 | 42 | 2 |
|  | Lecithin:cholesterol acyltransferase family protein, expressed | Os03g52010 | Q851F0_ORYSA | 2 | 78 | 5 |
|  | pentatricopeptide, putative, expressed | Os04g46010 | Q7XN02_ORYSA | 1 | 48 | 3 |
|  | Chlorophyll a-b binding protein 8, chloroplast precursor, putative, expressed | Os02g10390 | Q6H746_ORYSA | 1 | 42 | 9 |
| **6** | **Protein kinase domain containing protein** | **Os09g18010** | Q6ERY7_ORYSA | 1 | 81 | 1 |
|  | ATP-dependent Clp protease proteolytic subunit, putative, expressed | Os05g51450 | Q6L4S1_ORYSA | 1 | 50 | 3 |
| **7** | **Brassinosteroid insensitive1-associated receptor kinase 1 precursor, putative, expressed** | **Os06g16330** | Q9FP13_ORYSA | 6 | 473 | 13 |
|  | Heat shock cognate 70 kD protein 2, putative, expressed | Os03g60620 | Q84TA1_ORYSA | 4 | 246 | 6 |
|  | Chaperone protein dnaJ, putative, expressed | Os05g26902 | Q5W730_ORYSA | 1 | 48 | 2 |
| **8** | **Brassinosteroid insensitive 1-associated receptor kinase 1 precursor, putative, expressed** | **Os04g38480** | Q5Y8C8_ORYSA | 8 | 687 | 22 |
|  | NB-ARC domain containing protein, expressed | Os08g07890 | Q5KS46_ORYSA | 2 | 150 | 4 |
| **9** | **Leucine-rich repeat transmembrane protein kinase** | **Os07g48310** | Q7XHW7_ORYSA | 2 | 183 | 4 |
|  | Zinc finger, C2H2 type family protein, expressed | Os11g25610 | AAX96267 | 1 | 45 | 1 |
| **10** | **Putative leucine-rich repeat transmembrane protein** | **Os03g08550** | Q94HG1_ORYSA | 15 | 1193 | 32 |
|  | RNA recognition motif putative | Os02g12850 | Q6YVH3_ORYSA | 1 | 61 | 3 |
|  | Hypothetical protein | Os10g21540 | Q8W2W3_ORYSA | 1 | 44 | 2 |
|  | Serine carboxypeptidase | Os01g43890 | Q9FP87_ORYSA | 1 | 40 | 1 |
|  | ICE-like protease (caspase) p20 domain | Os01g58580 | Q8LJ88_ORYSA | 1 | 112 | 3 |
|  | Copper amine oxidase, enzyme domain, putative | Os07g38440 | Q69UD6_ORYSA | 1 | 41 | 5 |
| **11** | **Leucine-rich repeat transmembrane protein kinase** | **Os03g57780** | Q852J5_ORYSA | 6 | 373 | 8 |
|  | Stromal 70 kD heat shock-related protein, chloroplast precursor, putative, expressed | Os12g14070 | Q84P99_ORYSA | 2 | 141 | 11 |
|  | Zinc finger, C2H2 type family protein, expressed | Os11g25610 | AAX96267 | 1 | 45 | 1 |
| **12** | **Protein kinase domain, putative** | **Os01g60280** | Q5QMM2_ORYSA | 1 | 62 | 2 |
|  | dnaK-type molecular chaperone hsp70 | Os11g47760 | Q40693_ORYSA | 15 | 972 | 35 |
|  | dnaK protein | Os05g38530 | Q6L509_ORYSA | 3 | 211 | 6 |
| **13** | **Leucine Rich Repeat family protein, expressed** | **Os10g06760** | Q7XGP4_ORYSA | 4 | 388 | 7 |
|  | Stromal 70 kD heat shock-related protein, chloroplast precursor, putative, expressed | Os12g14070 | Q84P99_ORYSA | 1 | 54 | 6 |
|  | Serine carboxypeptidase family protein, expressed | Os01g43890 | Q9FP87_ORYSA | 1 | 41 | 1 |
| **14** | **Leucine Rich Repeat family protein, expressed** | **Os02g05950** | Q66QA6_ORYSA | 1 | 52 | 1 |
|  | NB-ARC domain containing protein | Os11g24170 | AAX96627 | 1 | 42 | <1 |
|  | Protein kinase domain containing protein | Os04g01890 | Q7XSW3_ORYSA | 1 | 44 | 1 |
|  | Protein kinase domain containing protein, expressed | Os07g35700 | Q84S59_ORYSA | 1 | 48 | 3 |
| **15** | **Protein kinase domain containing protein, expressed** | **Os02g53720** | Q6ZGC7_ORYSA | 1 | 65 | 3 |
|  | Expressed protein | Os01g68720 | Q8RZX3_ORYSA | 1 | 42 | 8 |
| **16** | **Leucine Rich Repeat family protein** | **Os01g53920** | Q5JKV7_ORYSA | 8 | 656 | 10 |
|  | Serine hydroxymethyltransferase, mitochondrial precursor, putative, expressed | Os03g52840 | Q7Y1F0_ORYSA | 1 | 93 | 3 |
|  | Aldehyde dehydrogenase, mitochondrial precursor, putative, expressed | Os02g49720 | Q6YWQ9_ORYSA | 1 | 65 | 3 |
|  | Alanine aminotransferase 2, putative, expressed | Os07g01760 | Q69UU3_ORYSA | 1 | 90 | 4 |
|  | Actin-1, putative, expressed | Os03g50890 | ATRZ1 | 1 | 96 | 4 |
|  | Tha8, putative, expressed | Os01g06480 | Q9FYP8_ORYSA | 1 | 39 | 2 |
|  | N-acetyl-gamma-glutamyl-phosphate reductase, chloroplast precursor, putative, expressed | Os03g42110 | Q6AV34_ORYSA | 1 | 45 | 2 |
|  | Eukaryotic initiation factor 4A, putative, expressed | Os06g48750 | Q5Z847_ORYSA | 1 | 44 | 3 |
|  | mRNA-binding protein precursor, putative, expressed | Os07g11110 | Q8GTK8_ORYSA | 1 | 97 | 4 |
|  | Cysteine synthase, chloroplast precursor, putative, expressed | Os01g74650 | Q5JNB0_ORYSA | 1 | 75 | 3 |
|  | Annexin family protein, expressed | Os05g31750 | Q6L4C6_ORYSA | 1 | 54 | 3 |
|  | Chlorophyll a-b binding protein 8, chloroplast precursor, putative, expressed | Os02g10390 | Q6H746_ORYSA | 1 | 55 | 9 |
|  | Ribonuclease T2 family protein, expressed | Os09g36680 | Q8RYA7_ORYSA | 1 | 54 | 5 |
|  | 2-cys peroxiredoxin BAS1, chloroplast precursor, putative, expressed | Os02g33450 | Q6ER94_ORYSA | 2 | 111 | 12 |
|  | CBS domain containing protein, expressed | Os03g52690 | Q84R32_ORYSA | 1 | 46 | 9 |
|  | Photosystem I reaction center subunit III, chloroplast precursor, putative,expressed | Os03g56670 | Q8S7H8_ORYSA | 1 | 41 | 7 |
|  | heavy metal-associated domain containing protein, expressed | Os05g27100 | Q5WMQ0_ORYSA | 1 | 136 | 4 |
| **17** | **Protein kinase, putative, expressed** | **Os01g12720** | Q8W0B8_ORYSA | 11 | 901 | 31 |
|  | Putative HSP70 | Os01g62290 | Q943K7_ORYSA | 5 | 352 | 14 |
|  | ribosomal protein S2 | Os10g38310 | Q5N985_ORYSA | 3 | 220 | 15 |
|  | Hypothetical protein | Os04g56430 | Q7X8N1_ORYSA | 1 | 82 | 5 |
| **18** | **Protein kinase domain containing protein, expressed** | **Os09g03620** | Q6K270_ORYSA | 10 | 764 | 22 |
|  | NB-ARC domain containing protein | Os11g24170 | AAX96627 | 1 | 42 | <1 |
| **19** | **Serine/threonine-protein kinase RLCKVII, putative, expressed** | **Os01g15470** | Q5NAV8_ORYSA | 3 | 243 | 2 |
|  | Heat shock cognate 70 kD protein, putative, expressed | Os11g47760 | Q40693_ORYSA | 7 | 455 | 15 |
|  | RuBisCO subunit binding-protein beta subunit, chloroplast precursor, putative, | Os06g02380 | Q9LWT6_ORYSA | 2 | 117 | 4 |
| **20** | **Serine/threonine-protein kinase NAK** | **Os10g26520** | Q7XEV8_ORYSA | 7 | 447 | 15 |
|  | Leucine-rich repeat transmembrane protein kinase, putative, expressed | Os05g40270 | Q5KQI5_ORYSA | 1 | 48 | 1 |
| **21** | **Protein kinase APK1B, chloroplast precursor, putative, expressed** | **Os06g45280** | Q654A0_ORYSA | 14 | 906 | 37 |
|  | Heat shock cognate 70 kD protein, putative, expressed | Os11g47760 | S53126 | 11 | 683 | 20 |
| **22** | **Protein kinase domain containing protein, expressed** | **Os04g41310** | Q7X6Q8_ORYSA | 3 | 209 | 14 |
|  | Leucine Rich Repeat family protein, expressed | Os02g05910 | Q5UD34_ORYRU | 1 | 82 | 2 |
| **23** | **Lectin protein kinase family protein** | **Os03g62180** | Q851M7_ORYSA | 11 | 1007 | 17 |
|  | Heat shock cognate 70 kD protein, putative, expressed | Os11g47760 | S53126 | 11 | 817 | 24 |
|  | RuBisCO subunit binding-protein beta subunit, chloroplast precursor, putative, expressed | Os06g02380 | Q9LWT6_ORYSA | 6 | 536 | 16 |
|  | RuBisCO subunit binding-protein alpha subunit, chloroplast precursor, putative, expressed | Os03g64210 | Q7X9A7_ORYSA | 1 | 91 | 2 |
|  | Cysteine protease 1 precursor, putative, expressed | Os04g57440 | KHRZOA | 1 | 77 | 2 |
| **24** | **D-mannose binding lectin family protein** | **Os04g01310** | Q7XMR2_ORYSA | 9 | 633 | 11 |
|  | Heat shock cognate 70 kD protein, putative, expressed | Os11g47760 | S53126 | 8 | 544 | 15 |
|  | Stromal 70 kD heat shock-related protein, chloroplast precursor, putative, expressed | Os12g14070 | Q84P99_ORYSA | 1 | 94 | 4 |
|  | RuBisCO subunit binding-protein alpha subunit, chloroplast precursor, putative, expressed | Os03g64210 | Q7X9A7_ORYSA | 2 | 233 | 5 |
|  | RuBisCO subunit binding-protein beta subunit, chloroplast precursor, putative, expressed | Os06g02380 | Q9LWT6_ORYSA | 3 | 183 | 8 |
|  | S3 self-incompatibility locus-linked pollen 3.15 protein, putative, expressed | Os03g45770 | Q75GP2_ORYSA | 1 | 51 | 1 |
|  | SHR5-receptor-like kinase, putative, expressed | Os05g16824 | Q7XTP3_ORYSA | 1 | 48 | 1 |
|  | Nucleosome assembly protein containing protein, expressed | Os02g36710 | Q69JW2_ORYSA | 1 | 94 | 7 |
| **25** | **OsMPK7 - putative MAPK based on amino acid sequence homology, expressed** | **Os06g48590** | Q5Z859_ORYSA | 4 | 204 | 11 |
|  | Eukaryotic initiation factor 4A, putative, expressed | Os06g48750 | Q5Z847_ORYSA | 2 | 97 | 5 |
|  | Chlorophyll a-b binding protein 8, chloroplast precursor, putative, expressed | Os02g10390 | Q6H746_ORYSA | 1 | 81 | 9 |
|  | Carbonic anhydrase, chloroplast precursor, putative, expressed | Os01g45274 | T03254 | 1 | 68 | 4 |
|  | CBS domain containing protein, expressed | Os08g22149 | Q6UUK6_ORYSA | 1 | 85 | 7 |
|  | 2-cys peroxiredoxin BAS1, chloroplast precursor, putative, expressed | Os02g33450 | Q6ER94_ORYSA | 1 | 45 | 5 |
|  | CBS domain containing protein, expressed | Os09g02710 | Q6YYV0_ORYSA | 1 | 73 | 6 |
|  | CBS domain containing protein, expressed | Os03g52690 | Q84R32_ORYSA | 1 | 70 | 9 |
|  | Histone H2A variant, putative, expressed | Os10g28230 | Q7XEK5_ORYSA | 1 | 62 | 6 |
| **26** | **OsMPK20-3 - putative MAPK based on amino acid sequence homology, expressed** | **Os06g26340** | Q5VN19_ORYSA | 1 | 51 | 1 |
|  | RuBisCO subunit binding-protein beta subunit, chloroplast precursor, putative, expressed | Os06g02380 | Q9LWT6_ORYSA | 5 | 456 | 14 |
|  | NmrA-like family protein, expressed | Os06g25439 | Q69SX2_ORYSA | 1 | 44 | 4 |
| **27** | **OsMPK20-5 - putative MAPK based on amino acid sequence homology, expressed** | **Os05g49140** | Q67C40_ORYSA | 6 | 394 | 13 |
|  | Retrotransposon protein, putative, unclassified | Os06g31530 | Q6ZFS9_ORYSA | 1 | 40 | 5 |
| **28** | **OsMPK20-4 - putative MAPK based on amino acid sequence homology, expressed** | **Os01g47530** | Q5SN53_ORYSA | 1 | 43 | 2 |
|  | Protein kinase domain, putative | Os05g40180 | Q75GM2_ORYSA | 1 | 45 | 4 |
| **29** | **Tousled-like kinase 2, putative, expressed** | **Os03g53880** | Q8W2Z0_ORYSA | 2 | 151 | 2 |
|  | Vacuolar ATP synthase catalytic subunit A, putative, expressed | Os06g45120 | Q651T8_ORYSA | 1 | 105 | 1 |
| **30** | **Protein kinase G11A, putative, expressed** | **Os09g30150** | Q651X1_ORYSA | 11 | 955 | 25 |
|  | Stromal 70 kD heat shock-related protein, chloroplast precursor, putative, expressed | Os12g14070 | Q2QV45_ORYSA | 2 | 139 | 4 |
| **31** | **Protein kinase domain containing protein, expressed** | **Os06g08280** | Q5SMJ0_ORYSA | 11 | 818 | 11 |
|  | Potassium channel AKT2/3, putative, expressed | Os05g35410 | Q5TKJ4_ORYSA | 1 | 47 | 1 |
|  | Heat shock cognate 70 kD protein, putative, expressed | Os11g47760 | S53126 | 9 | 697 | 18 |
|  | ATP-dependent protease La domain containing protein, expressed | Os07g32560 | Q6Z4A9_ORYSA | 1 | 98 | 4 |
| **32** | **Protein kinase domain containing protein, expressed** | **Os09g33630** | Q69SG8_ORYSA | 1 | 81 | 22 |
|  | Heat shock cognate 70 kDa protein, putative, expressed | Os11g47760 | S53126 | 12 | 1681 | 44 |
|  | Heat shock cognate 70 kDa protein, putative, expressed | Os01g62290 | Q943K7_ORYSA | 13 | 1529 | 40 |
|  | Heat shock cognate 70 kDa protein, putative, expressed | Os05g38530 | Q6L509_ORYSA | 13 | 1523 | 42 |
|  | Chaperonin CPN60-1, mitochondrial precursor, putative, expressed | Os10g32550 | Q8H903_ORYSA | 3 | 140 | 10 |
|  | RuBisCO subunit binding-protein beta subunit, chloroplast precursor, putative, expressed | Os02g01280 | Q6ZFJ9_ORYSA | 1 | 47 | 2 |
|  | NB-ARC domain containing protein, expressed | Os10g33440 | Q7XDG5_ORYSA | 1 | 50 | <1 |
|  | Actin-1, putative, expressed | Os03g50890 | ATRZ1 | 1 | 112 | 4 |
|  | hypothetical protein | Os09g15580 | Q6K3X1_ORYSA | 1 | 107 | 4 |
|  | Protein kinase domain containing protein, expressed | Os02g37830 | Q6YY75_ORYSA | 2 | 54 | 4 |
|  | expressed protein | Os08g44000 | Q6Z3F9_ORYSA | 1 | 45 | 4 |

**C. TAP-tagged protein kinase purifications where either the tagged protein or associated protein(s) were not detected**

| S. No. | Protein Name | TIGR_id | SwissProt_id | No. of peptides | Score | % coverage |
| --- | --- | --- | --- | --- | --- | --- |
| **1** | **Holocarboxylase synthetase, putative** | **Os01g40690** | -- | **--** | **--** | **--** |
|  | Potassium channel AKT2/3, putative, expressed | Os05g35410 | Q5TKJ4_ORYSA | 1 | 43 | 1 |
| **2** | **Serine/threonine-protein kinase AtPK19, putative, expressed** | **Os03g21620** | -- | **--** | **--** | **--** |
| **3** | **serine/threonine-protein kinase SAPK6, putative, expressed** | **Os02g34600** | Q6ZI44_ORYSA | 6 | 468 | 21 |
| **4** | **Calcium-dependent protein kinase, isoform 11, putative, expressed** | **Os04g49510** | Q9FXQ3_ORYSA | 15 | 1239 | 33 |
| **5** | **Serine/threonine-protein kinase 12, putative, expressed** | **Os03g55620** | Q7Y0C4_ORYSA | 1 | 48 | 5 |
| **6** | **Protein kinase domain containing protein, expressed** | **Os03g16130** | -- | **--** | **--** | **--** |
| **7** | **Serine/threonine-protein kinase MHK, putative, expressed** | **Os03g63020** | -- | **--** | **--** | **--** |
| **8** | **Transposon protein, unclassified** | **Os01g27020** | -- | **--** | **--** | **--** |
|  | zinc finger family protein, putative, expressed | Os05g01230 | Q9FW25_ORYSA | 1 | 46 | 1 |
| **9** | **Protein kinase family protein, putative, expressed** | **Os01g54100** | -- | **--** | **--** | **--** |
|  | NB-ARC domain containing protein | Os11g24170 | AAX96627 | 1 | 42 | <1 |
|  | 28 kD ribonucleoprotein, chloroplast, putative, expressed | Os09g39180 | Q650W6_ORYSA | 1 | 49 | 3 |
|  | zinc finger family protein, putative, expressed | Os05g01230 | Q9FW25_ORYSA | 1 | 43 | 1 |
|  | Serine carboxypeptidase family protein, expressed | Os01g43890 | Q9FP87_ORYSA | 1 | 41 | 1 |
| **10** | **Protein kinase domain containing protein** | **Os05g25370** | -- | **--** | **--** | **--** |
|  | Hypothetical protein | Os03g60660 | Q84TB1_ORYSA | 1 | 48 | 7 |
|  | LEC14B protein, putative, expressed | Os05g33710 | Q6L548_ORYSA | 1 | 40 | 3 |
| **11** | **Lectin receptor kinase 7, putative, expressed** | **Os07g03820** | -- | **--** | **--** | **--** |
| **12** | **Putative receptor serine/threonine kinase** | **Os01g04409** | -- | **--** | **--** | **--** |
| **13** | **Protein kinase domain, putative** | **Os01g48390** | Q5QM36_ORYSA | 9 | 693 | 34 |
| **14** | **Protein kinase domain containing protein** | **Os05g48660** | -- | **--** | **--** | **--** |
| **15** | **SHR5-receptor-like kinase, putative, expressed** | **Os05g16740** | Q6F2P3_ORYSA | 1 | 88 | 1 |
| **16** | **Protein kinase domain containing protein, expressed** | **Os06g46330** | Q653V5_ORYSA | 8 | 556 | 25 |
| **17** | **Protein kinase domain containing protein, expressed** | **Os03g56470** | -- | **--** | **--** | **--** |
|  | hypothetical protein | Os10g21540 | Q8W2W3_ORYSA | 1 | 44 | 2 |
|  | hypothetical protein | Os01g68720 | Q8RZX3_ORYSA | 1 | 49 | 8 |
| **18** | **Protein kinase APK1B, chloroplast precursor** | **Os08g35600** | -- | **--** | **--** | **--** |
| **19** | **Serine/threonine-protein kinase RLCKVII, putative, expressed** | **Os03g03880** | -- | **--** | **--** | **--** |
|  | NB-ARC domain containing protein | Os11g24170 | AAX96627 | 1 | 39 | >1 |
| **20** | **Serine/threonine-protein kinase RLCKVII, putative, expressed** | **Os05g30870** | -- | **--** | **--** | **--** |
|  | Heat shock 70 kD protein, mitochondrial precursor, putative, expressed | Os02g53420 | Q6Z7L1_ORYSA | 2 | 93 | 4 |
|  | Chaperonin CPN60-1, mitochondrial precursor, putative, expressed | Os10g32550 | Q8H903_ORYSA | 1 | 110 | 4 |
| **21** | **Serine/threonine-protein kinase NAK, putative, expressed** | **Os03g29410** | -- | **--** | **--** | **--** |
|  | Hypothetical protein | Os11g12610 | Q60EE3_ORYSA | 1 | 43 | 5 |
|  | Expressed protein | Os06g18960 | Q5Z567_ORYSA | 1 | 41 | 1 |
|  | CBS domain containing protein, expressed | Os02g06410 | Q6ETI3_ORYSA | 1 | 45 | 2 |
| **22** | **Protein kinase domain containing protein, expressed** | **Os03g62700** | -- | **--** | **--** | **--** |
| **23** | **Protein kinase domain containing protein, expressed** | **Os05g04520** | -- | **--** | **--** | **--** |
| **24** | **Protein kinase domain containing protein, expressed** | **Os02g02040** | -- | **--** | **--** | **--** |
| **25** | **U-box domain containing protein, expressed** | **Os09g39640** | -- | **--** | **--** | **--** |
| **26** | **U-box domain containing protein** | **Os06g04880** | -- | **--** | **--** | **--** |
|  | LEC14B protein, putative, expressed | Os05g33710 | Q6L548_ORYSA | 1 | 40 | 3 |
| **27** | **Protein kinase domain containing protein, expressed** | **Os02g09359** | -- | **--** | **--** | **--** |
|  | Zinc finger, C2H2 type family protein, expressed | Os11g25610 | AAX96267 | 1 | 46 | 1 |
| **28** | **S-locus glycoprotein family protein, expressed** | **Os05g42210** | Q60EJ2_ORYSA | 7 | 548 | 10 |
| **29** | **D-mannose binding lectin family protein, expressed** | **Os12g34320** | -- | **--** | **--** | **--** |
| **30** | **D-mannose binding lectin family protein, expressed** | **Os01g65030** | -- | **--** | **--** | **--** |
| **31** | **Calcium binding EGF domain containing protein, expressed** | **Os04g20680** | -- | **--** | **--** | **--** |
| **32** | **CIPK-like protein 1, putative, expressed** | **Os03g22050** | -- | **--** | **--** | **--** |
| **33** | **CBL-interacting serine/threonine-protein kinase 1, putative, expressed** | **Os05g04550** | -- | **--** | **--** | **--** |
|  | RuBisCO subunit binding-protein beta subunit, chloroplast precursor, putative, expressed | Os06g02380 | Q9LWT6_ORYSA | 5 | 482 | 12 |
|  | RuBisCO subunit binding-protein alpha subunit, chloroplast precursor, putative, expressed | Os03g64210 | Q7X9A7_ORYSA | 3 | 303 | 7 |
| **34** | **Protein kinase domain containing protein** | **Os01g43910** | -- | **--** | **--** | **--** |
| **35** | **Protein kinase domain containing protein** | **Os06g49430** | -- | **--** | **--** | **--** |
|  | Serine hydroxymethyltransferase, mitochondrial precursor, putative, expressed | Os03g52840 | Q7Y1F0_ORYSA | 1 | 97 | 3 |
|  | NB-ARC domain containing protein | Os11g24170 | AAX96627 | 1 | 42 | <1 |
|  | DREPP plasma membrane polypeptide family protein, expressed | Os01g13210 | BAA96588 | 1 | 39 | 7 |
|  | Chlorophyll a-b binding protein 8, chloroplast precursor, putative, expressed | Os02g10390 | Q6H746_ORYSA | 1 | 71 | 9 |
|  | CBS domain containing protein, expressed | Os09g02710 | Q6YYV0_ORYSA | 1 | 71 | 6 |
|  | CBS domain containing protein, expressed | Os03g52690 | Q84R32_ORYSA | 1 | 46 | 9 |
|  | heavy metal-associated domain containing protein, expressed | Os05g27100 | Q5WMQ0_ORYSA | 1 | 150 | 4 |
| **36** | **Extra-cellular signal-regulated kinase 1, putative, expressed** | **Os11g17080** | -- | **--** | **--** | **--** |
|  | Protein phosphatase 2C containing protein, expressed | Os06g39600 | Q69VD8_ORYSA | 1 | 44 | 2 |
| **37** | **Protein kinase G11A, putative, expressed** | **Os02g49310** | **--** | **--** | **--** | **--** |
| **38** | **Protein kinase domain containing protein** | **Os07g29330** | -- | **--** | **--** | **--** |
| **39** | **Protein kinase domain containing protein** | **Os06g12590** | Q67UL6_ORYSA | 1 | 84 | 2 |
| **40** | **Protein kinase domain containing protein** | **Os08g01270** | -- | **--** | **--** | **--** |
| **41** | **Protein kinase domain containing protein, expressed** | **Os02g38080** | Q6ZH81_ORYSA | 4 | 241 | 18 |
| **42** | **Protein kinase domain containing protein, expressed** | **Os02g39560** | Q6K1X9_ORYSA | 4 | 382 | 11 |
| **43** | **Protein kinase family protein** | **Os06g29120** | Q67WX3_ORYSA | 1 | 105 | 2 |

-- no peptide recovered for that particular protein
