# Supplementary material for: Protein-Protein Interactions of Tandem Affinity Purified Protein Kinases from Rice
Source: PLoS One. 2009 Aug 19;4(8):e6685. doi: 10.1371/journal.pone.0006685 (PMC2723914; doi:10.1371/journal.pone.0006685)
Supplement: Table S2 — Proteins subtracted from the protein-protein interaction data base as recurring/contaminant proteins (0.06 MB DOC) [file pone.0006685.s002.doc]

**Supplementary Table S2. Proteins subtracted from the protein-protein interaction data base as recurring/ contaminant proteins (from 129 Tandem affinity purifications):**

**Ribosomal Proteins (120):**

60S ribosomal protein L2, putative, expressed (Os12g38000); 60S ribosomal protein L3, putative, expressed (Os11g06750); 60S ribosomal protein L3, putative, expressed (Os12g07010); 60S ribosomal protein L4, putative, expressed (Os07g08330);

60S ribosomal protein L5-1, putative, expressed (Os01g67134); 60S ribosomal protein L6, putative, expressed (Os02g37862); 60S ribosomal protein L6, putative, expressed (Os04g39700); 60S ribosomal protein L7-1, putative, expressed (Os04g51630);

60S ribosomal protein L7-1, putative, expressed (Os08g13690); 60S ribosomal protein L7a, putative, expressed (Os08g23710); 60S ribosomal protein L9, putative, expressed (Os02g01332); 60S ribosomal protein L10a-1, putative, expressed (Os01g64090);

60S ribosomal protein L10a-1, putative, expressed (Os02g21660); 60S ribosomal protein L10a-1, putative, expressed (Os08g44380); 60S ribosomal protein L10a-1, putative, expressed (Os08g44450); 60S ribosomal protein L11, putative, expressed (Os01g10820);

60S ribosomal protein L12, putative, expressed (Os02g47140); 60S ribosomal protein L13-2, putative, expressed (Os03g37970); 60S ribosomal protein L13-2, putative, expressed (Os06g02510); 60S ribosomal protein L13a-2, putative, expressed (Os03g54890); 60S ribosomal protein L13a-2, putative, expressed (Os07g01870);

60 ribosomal protein L14, putative, expressed (Os02g40880); 60S ribosomal protein L17, putative, expressed (Os08g41810); 60S ribosomal protein L17, putative, expressed (Os09g08430); 60S ribosomal protein L18, putative, expressed (Os05g06310); 60S ribosomal protein L18, putative, expressed (Os07g47780); 60S ribosomal protein L18a, putative, expressed (Os01g54870); 60S ribosomal protein L18a, putative, expressed (Os05g49030); 60S ribosomal protein L19-3, putative, expressed (Os03g38260);

60S ribosomal protein L22-2, putative, expressed (Os03g22340); 60S ribosomal protein L23, putative, expressed (Os02g56960); 60S ribosomal protein L23A, putative, expressed (Os01g24690); 60S ribosomal protein L24, putative, expressed (Os01g59990); 60S ribosomal protein L24, putative, expressed (Os05g40820); 60S ribosomal protein L24, putative, expressed (Os07g12250); 60S ribosomal protein L26-1, putative, expressed (Os01g04730); 60S ribosomal protein L26-1, putative, expressed (Os11g05370); 60S ribosomal protein L26-1, putative, expressed (Os12g05430); 60S ribosomal protein L27, putative, expressed (Os02g18380); 60S ribosomal protein L27, putative, expressed (Os10g41470); 60S ribosomal protein L27a-2, putative, expressed (Os07g42170); 60S ribosomal protein L30, putative, expressed (Os01g16890); 60S ribosomal protein L32, putative, expressed (Os08g41300); 60S ribosomal protein L33-B, putative, expressed (Os02g54470); 60S ribosomal protein L33-B, putative, expressed (Os05g48220); 60S ribosomal protein L34, putative, expressed (Os08g06040); 60S ribosomal protein L35, putative, expressed (Os02g30050); 60S ribosomal protein L36-2, putative, expressed (Os01g62350); 60S ribosomal protein L36-2, putative, expressed (Os05g38520); 60S ribosomal protein L37a, putative, expressed (Os01g48770); 60s acidic ribosomal protein, expressed (Os05g37330); 60S acidic ribosomal protein P0, putative, expressed (Os08g03640); 60S acidic ribosomal protein P1, putative, expressed (Os08g02340); 60S acidic ribosomal protein P3, putative, expressed (Os01g13080); 60S acidic ribosomal protein P2A, putative, expressed (Os01g09510); 50S ribosomal protein L1, putative, expressed (Os05g32220); 50S ribosomal protein L3, chloroplast precursor, putative, expressed (Os02g04460); 50S ribosomal protein L4, chloroplast precursor, putative, expressed (Os03g15870); 50S ribosomal protein L5, chloroplast precursor, putative, expressed (Os03g03360); 50S ribosomal protein L6, putative, expressed (Os03g54040); 50S ribosomal protein L9, chloroplast precursor, putative, expressed (Os02g57670); 50S ribosomal protein L12-2, chloroplast precursor, putative, expressed (Os01g47330); 50S ribosomal protein L13, chloroplast precursor, putative, expressed (Os01g54540); 50S ribosomal protein L14, putative (Os01g57950); 50S ribosomal protein L17, putative, expressed (Os03g60100); 50S ribosomal protein L18, chloroplast precursor, putative, expressed (Os03g61260); 50S ribosomal protein L20, putative, expressed (Os05g45220); 50S ribosomal protein L21, chloroplast precursor, putative, expressed (Os02g15900); 50S ribosomal protein L24, chloroplast precursor, putative, expressed (Os06g46930); 50S ribosomal protein L27, chloroplast precursor, putative, expressed (Os01g69950); 50S ribosomal protein L29, chloroplast precursor, putative, expressed (Os02g51790); 40S ribosomal protein SA, putative, expressed (Os03g08440); 40S ribosomal protein S2, putative, expressed (Os03g59310); 40S ribosomal protein S2, putative, expressed (Os07g10660); 40S ribosomal protein S3, putative, expressed (Os03g38000); 40S ribosomal protein S3a, putative, expressed (Os03g10340); 40S ribosomal protein S3a, putative, expressed (Os02g18550); 40S ribosomal protein S3-A, putative, expressed (Os07g41750); 40S ribosomal protein S4, putative, expressed (Os01g25610); 40S ribosomal protein S4, putative, expressed (Os02g01560); 40S ribosomal protein S4, putative, expressed (Os05g30530); 40S ribosomal protein S5, putative, expressed (Os11g29190); 40S ribosomal protein S6, putative, expressed (Os07g42950); 40S ribosomal protein S6, putative, expressed (Os03g27260); 40S ribosomal protein S7, putative, expressed (Os05g27940); 40S ribosomal protein S8, putative, expressed (Os04g28180); 40S ribosomal protein S8, putative, expressed (Os02g28810); 40S ribosomal protein S9, putative, expressed (Os03g05980); 40S ribosomal protein S11, putative, expressed (Os04g52354); 40S ribosomal protein S12, putative, expressed (Os07g05580); 40S ribosomal protein S12, putative, expressed (Os07g12650); 40S ribosomal protein S13, putative, expressed (Os08g02410); 40S ribosomal protein S14, putative, expressed (Os02g06700); 40S ribosomal protein S15, putative, expressed (Os03g58430); 40S ribosomal protein S15a, putative, expressed (Os02g27760); 40S ribosomal protein S15a, putative, expressed (Os02g15610); 40S ribosomal protein S16, putative, expressed (Os11g03400); 40S ribosomal protein S17, putative, expressed (Os03g01900); 40S ribosomal protein S17-4, putative, expressed (Os10g27190); 40S ribosomal protein S18, putative, expressed (Os03g58050); 40S ribosomal protein S19, putative, expressed (Os03g31090); 40S ribosomal protein S20, putative, expressed (Os10g08930); 40S ribosomal protein S20, putative, expressed (Os06g04290); 40S ribosomal protein S24, putative, expressed (Os06g36160); 40S ribosomal protein S25, putative, expressed (Os08g44480); 40S ribosomal protein S26, putative, expressed (Os01g60790); 40S ribosomal protein S30, putative, expressed (Os06g07580); 30S ribosomal protein S9, chloroplast precursor, putative, expressed (Os03g55930); 30S ribosomal protein S13, putative, expressed (Os03g49710); Plastid-specific 30S ribosomal protein 1, chloroplast precursor, putative, expressed (Os03g63950); Plastid-specific 30S ribosomal protein 2, chloroplast precursor, putative, expressed (Os09g10760); Chloroplast 30S ribosomal protein S3, putative, expressed (Os04g16780); ribosomal protein S5 containing protein, expressed (Os03g34040); Ribosomal protein S12 containing protein (Os12g33930); ribosomal protein S15 containing protein (Os04g16720); ribosomal protein S18 containing protein, expressed (Os10g35690); ribosomal protein S21 containing protein, expressed (Os02g09590); ribosomal protein L10 containing protein, expressed (Os03g17580); ribosomal protein L19 containing protein, expressed (Os02g43600); Ribosomal L28e protein family protein, expressed (Os05g46430); Ribosomal L28e protein family protein, expressed (Os02g57540)

**Rubisco (5):**

Ribulose-phosphate 3-epimerase, chloroplast precursor, putative, expressed (Os03g07300); Ribulose bisphosphate carboxylase/oxygenase activase, chloroplast precursor, putative, expressed (Os11g47970); Ribulose bisphosphate carboxylase small chain A, chloroplast precursor, putative, expressed (Os12g19394); Ribulose bisphosphate carboxylase small chain C, chloroplast precursor, putative, expressed (Os12g17600); Ribulose bisphosphate carboxylase large chain precursor, putative, expressed (Os10g21280)

**Expressed proteins (4):**

expressed protein (Os02g07890); expressed protein (Os04g42210); expressed protein (Os05g37400); hypothetical protein (Os06g12930)

**Others (22):**

ATP synthase alpha chain, putative, expressed (Os04g16740); ATP synthase alpha chain, mitochondrial, putative, expressed (Os09g08910); ATP synthase beta chain, putative, expressed (Os10g21270); ATP synthase gamma chain, chloroplast precursor, putative, expressed (Os07g32880); Vacuolar ATP synthase catalytic subunit A, putative, expressed (Os02g07870); Vacuolar ATP synthase subunit B isoform 1, putative, expressed (Os06g37180); Vacuolar ATP synthase subunit G2, putative, expressed (Os04g51270); Elongation factor Tu, chloroplast precursor, putative, expressed (Os02g38210); Xyloglucan galactosyltransferase KATAMARI 1, putative, expressed (Os03g05110); Protochlorophyllide reductase A, chloroplast precursor, putative, expressed (Os04g58200); Protochlorophyllide reductase B, chloroplast precursor, putative, expressed (Os10g35370); plasma membrane intrinsic polypeptide, putative, expressed (Os02g18410); Luminal binding protein 3 precursor, putative, expressed (Os02g02410);

Luminal binding protein 5 precursor, putative, expressed (Os05g35400); Luminal binding protein 5 precursor, putative (Os08g09770); Glutamate decarboxylase, putative, expressed (Os03g51080); Glutamate decarboxylase, putative, expressed (Os08g36320);

Glyceraldehyde-3-phosphate dehydrogenase, cytosolic, putative, expressed (Os08g03290); Glyceraldehyde-3-phosphate dehydrogenase A, chloroplast precursor, putative, expressed (Os04g38600); Glyceraldehyde-3-phosphate dehydrogenase B, chloroplast precursor, putative, expressed (Os03g03720); Phosphoglycerate kinase, chloroplast precursor, putative, expressed (Os05g41640); Succinate dehydrogenase iron-sulfur protein,mitochondrial precursor, putative, expressed (Os08g02640)
