# Supplementary material for: Protein-Protein Interactions of Tandem Affinity Purified Protein Kinases from Rice
Source: PLoS One. 2009 Aug 19;4(8):e6685. doi: 10.1371/journal.pone.0006685 (PMC2723914; doi:10.1371/journal.pone.0006685)
Supplement: File S1 — Gel pictures of purifications that identified protein complexes. (0.11 MB PDF) [file pone.0006685.s005.pdf]

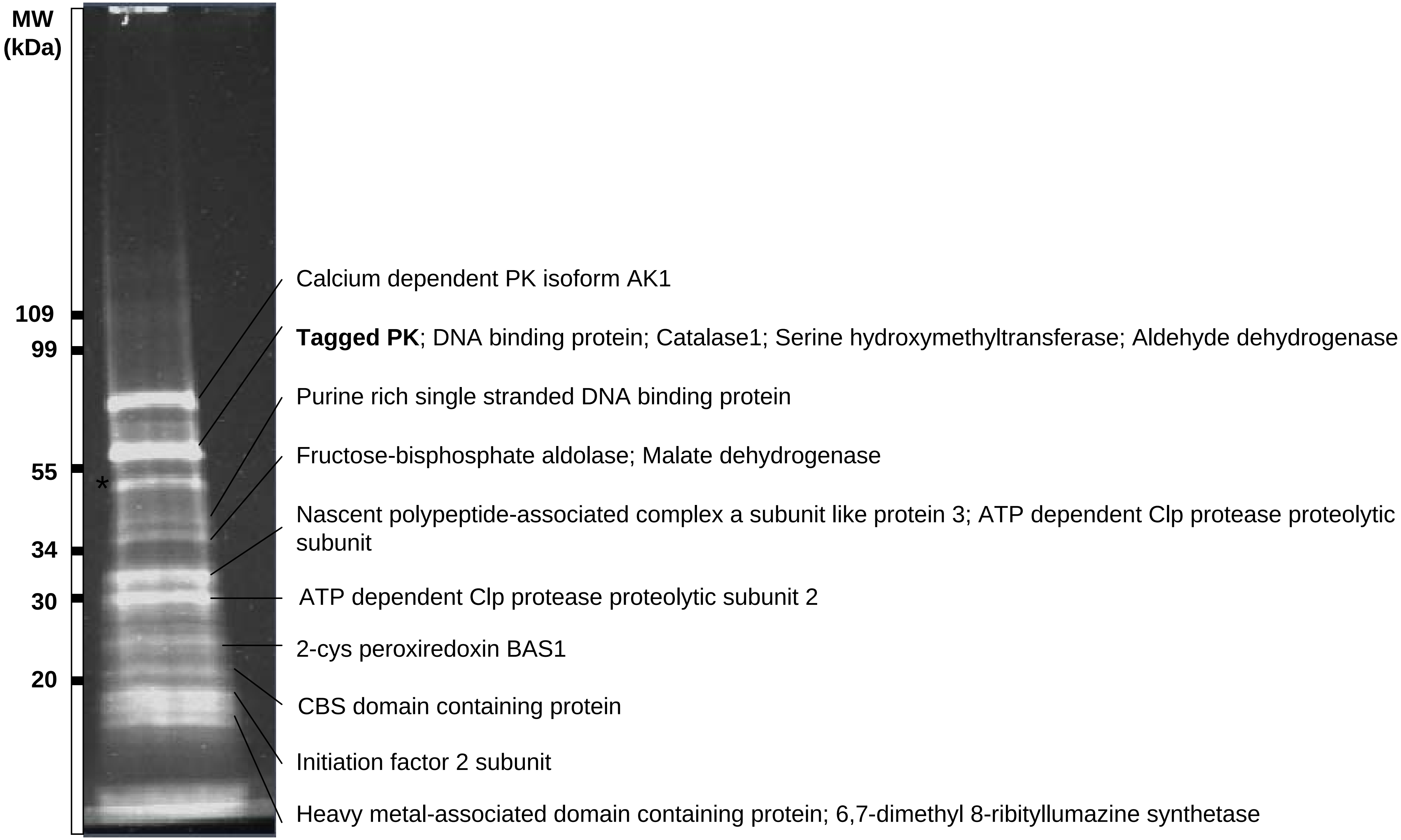

Table 1 # 1: Calcium-dependent protein kinase, isoform 11 (Os03g03660)

* Contaminant and low scoring tagged protein

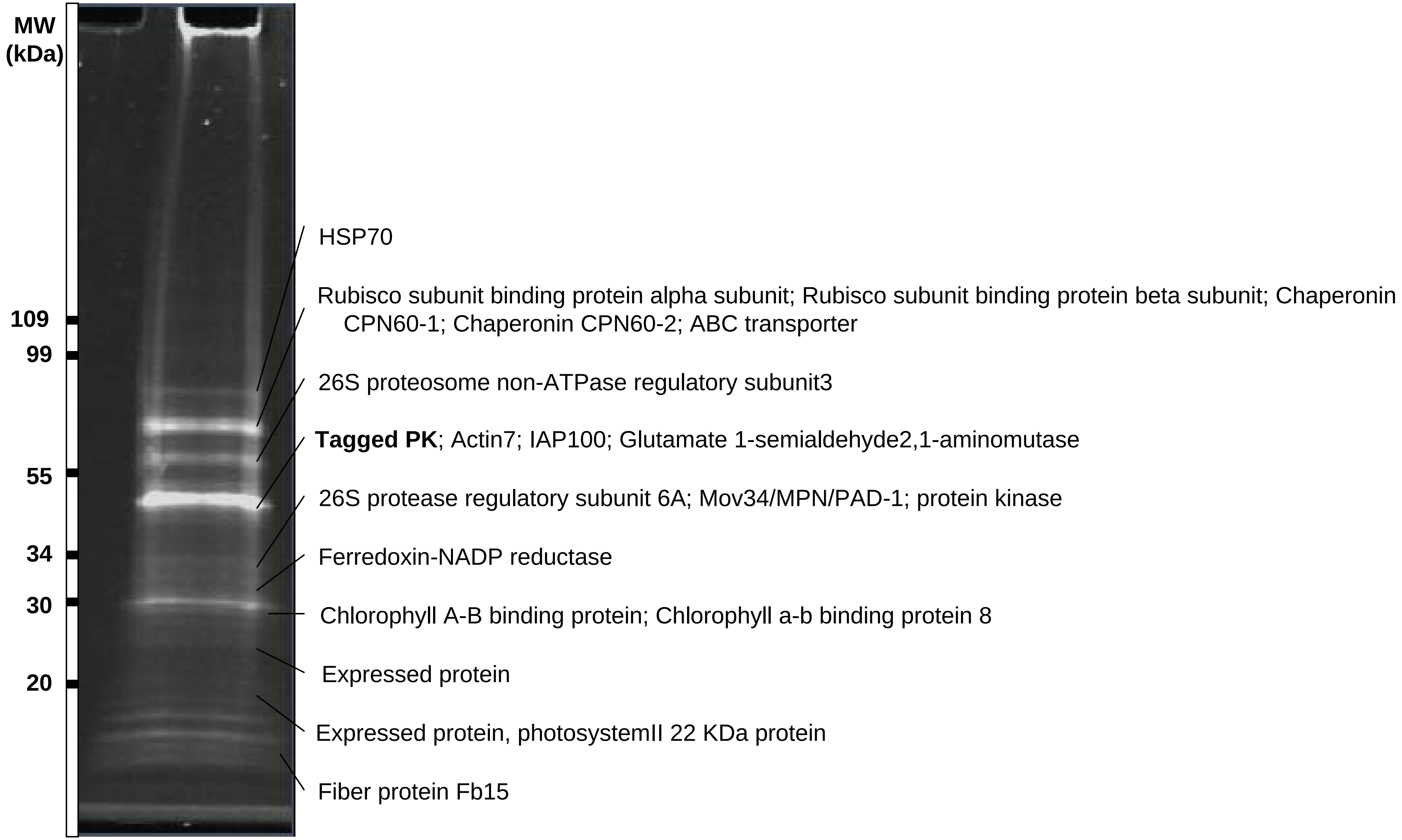

Table 1 # 2: Lectin receptor kinase 7 (Os07g38800)

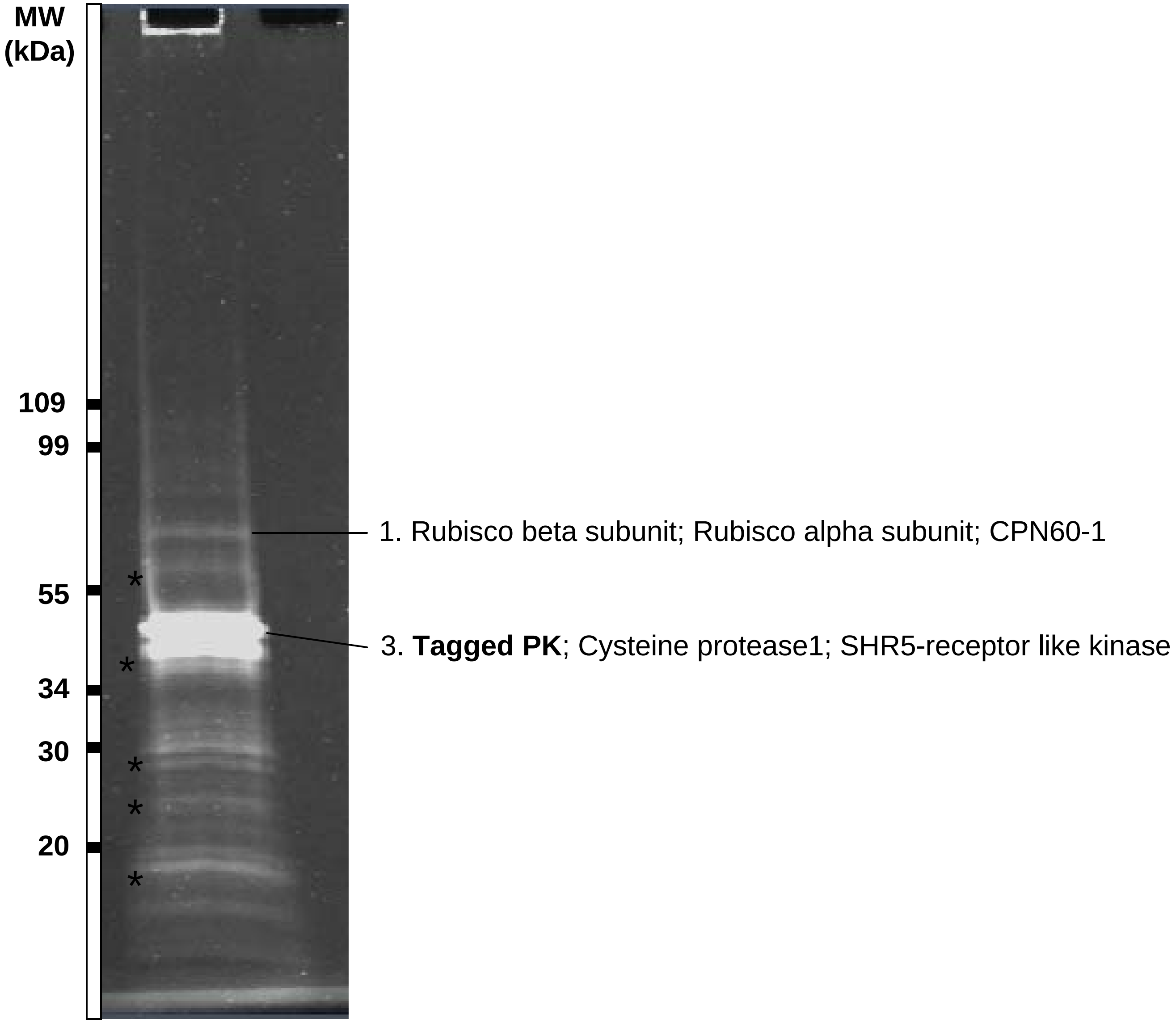

Table 1 # 3: Lectin protein kinase (Os07g38810)

* Contaminant and low scoring tagged protein

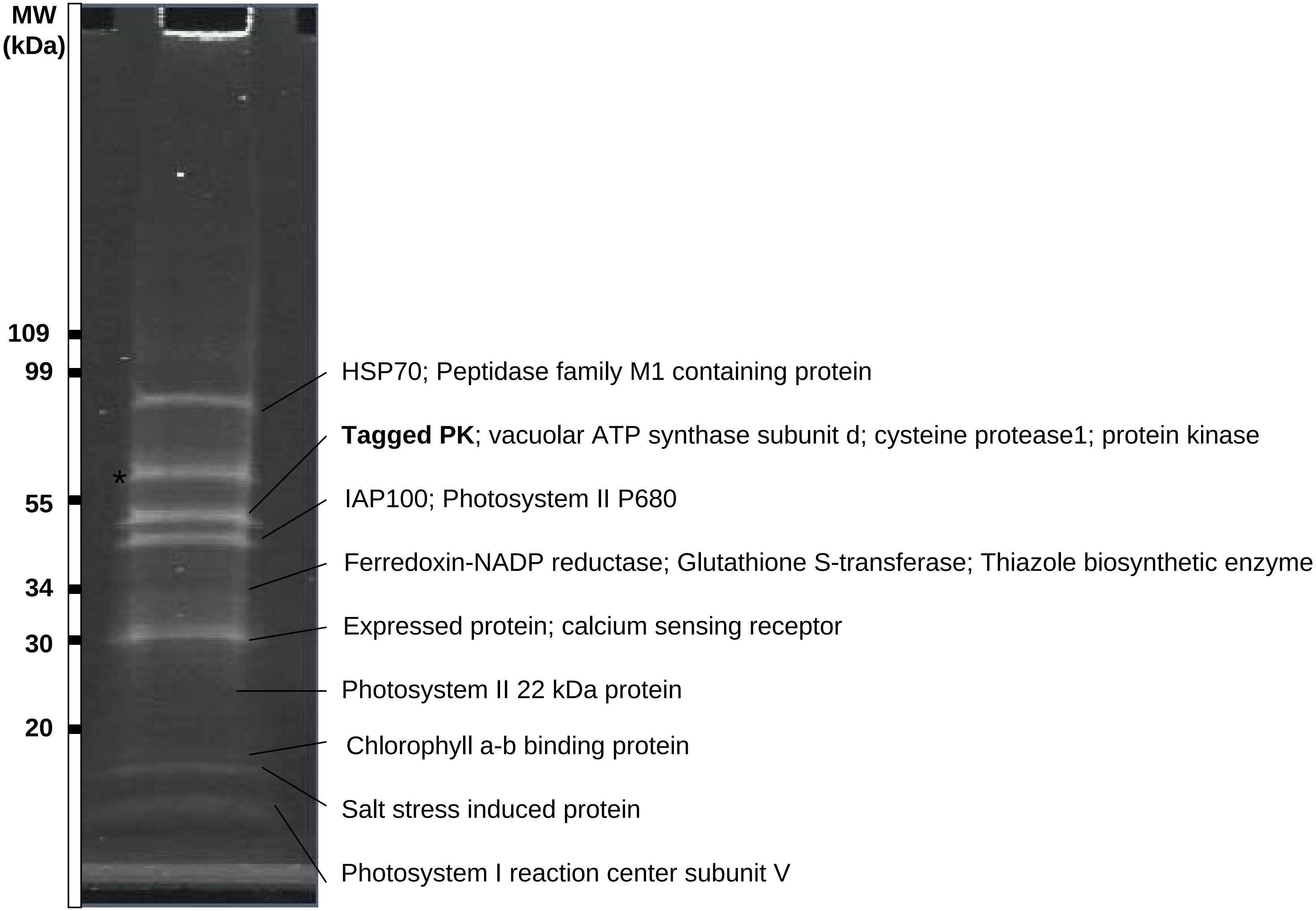

Table 1 # 4: Protein kinase domain containing protein (Os01g14510)

* Contaminant and low scoring tagged protein

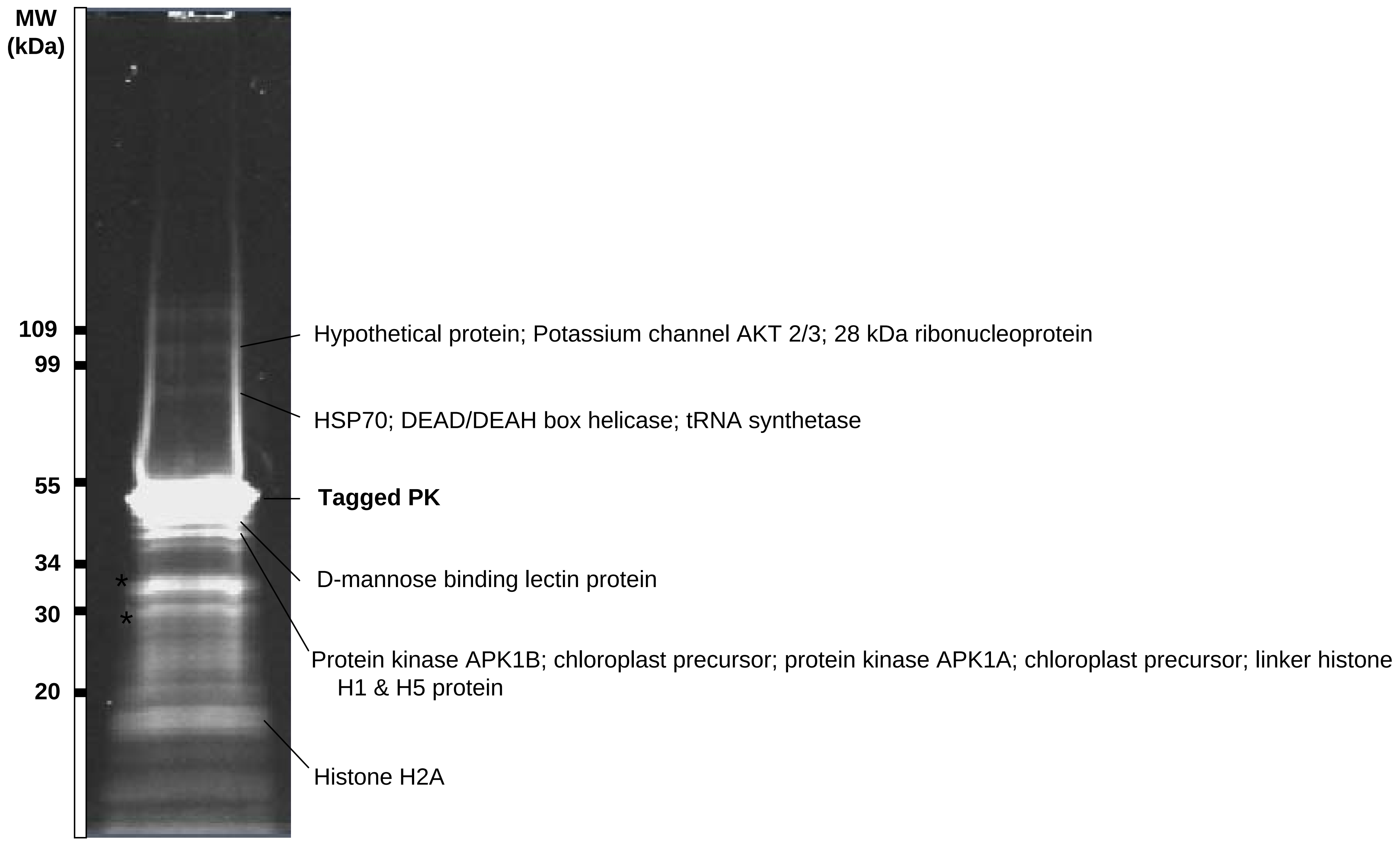

Table 1 # 5: Protein kinase APK1B (Os03g06330)

* Contaminant and low scoring tagged protein

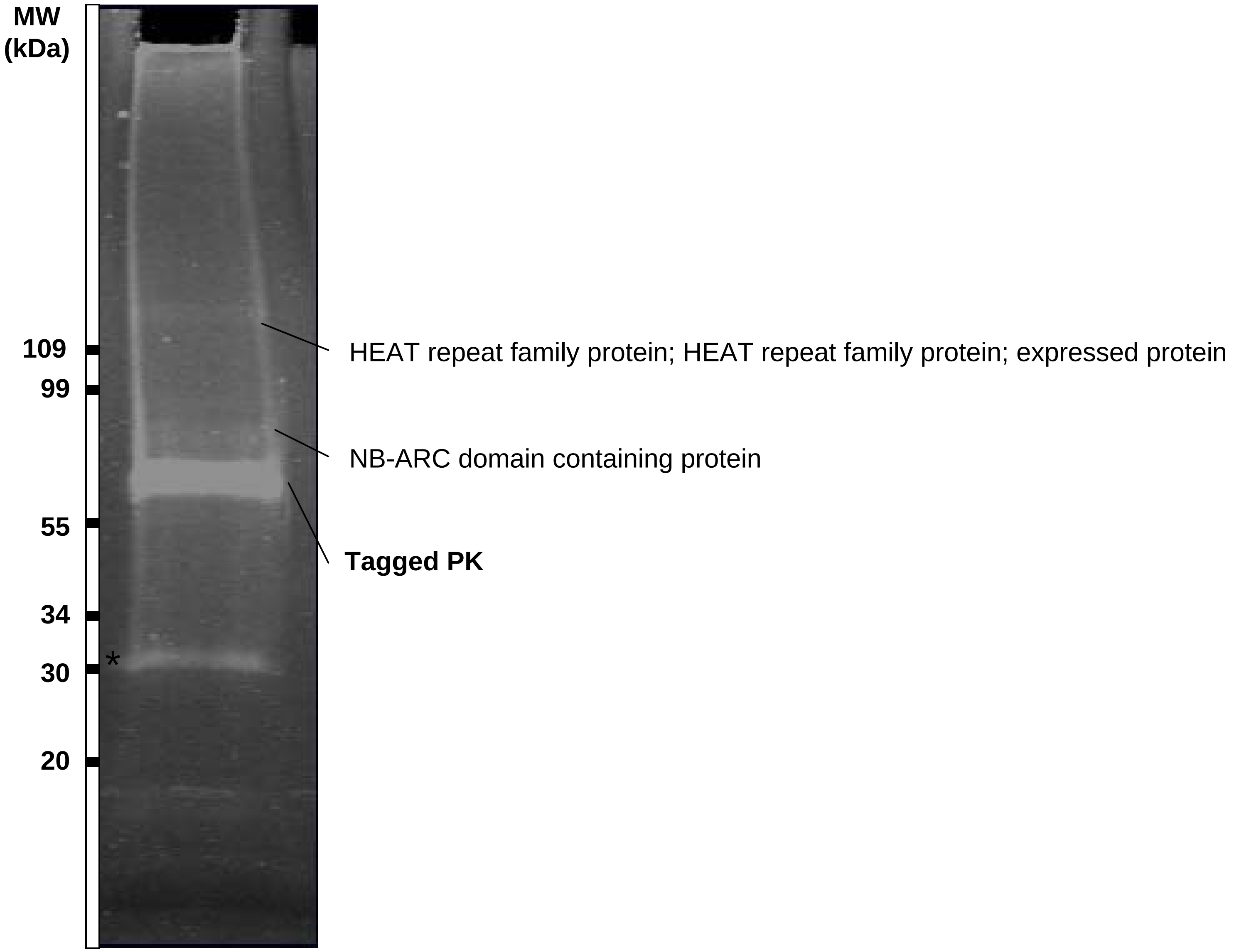

Table 1 # 6: Serine/threonine-protein kinase RLCKVII (Os07g49470)

* Contaminant and low scoring tagged protein

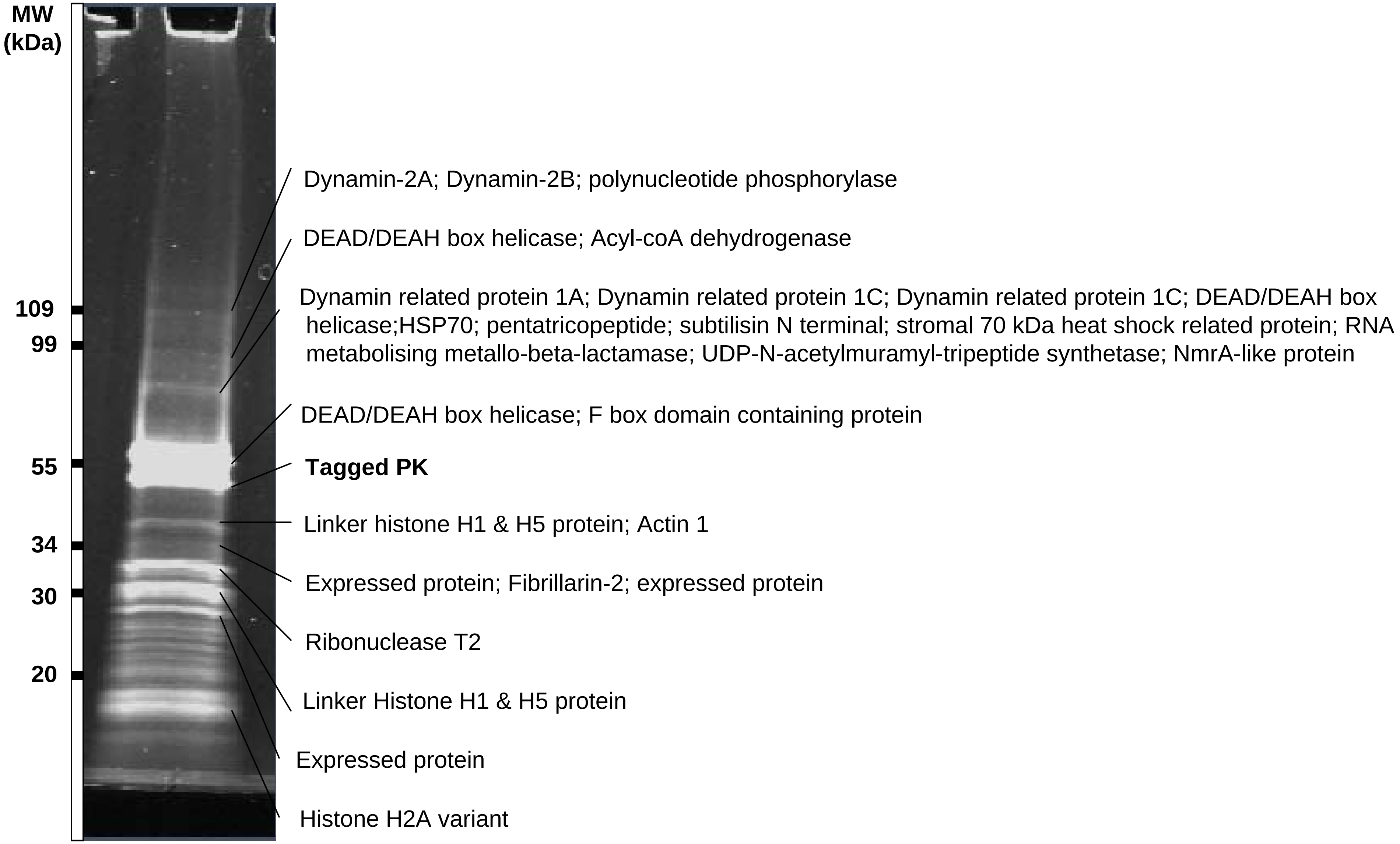

Table 1 # 7: Protein kinase APK1A (Os05g02020)

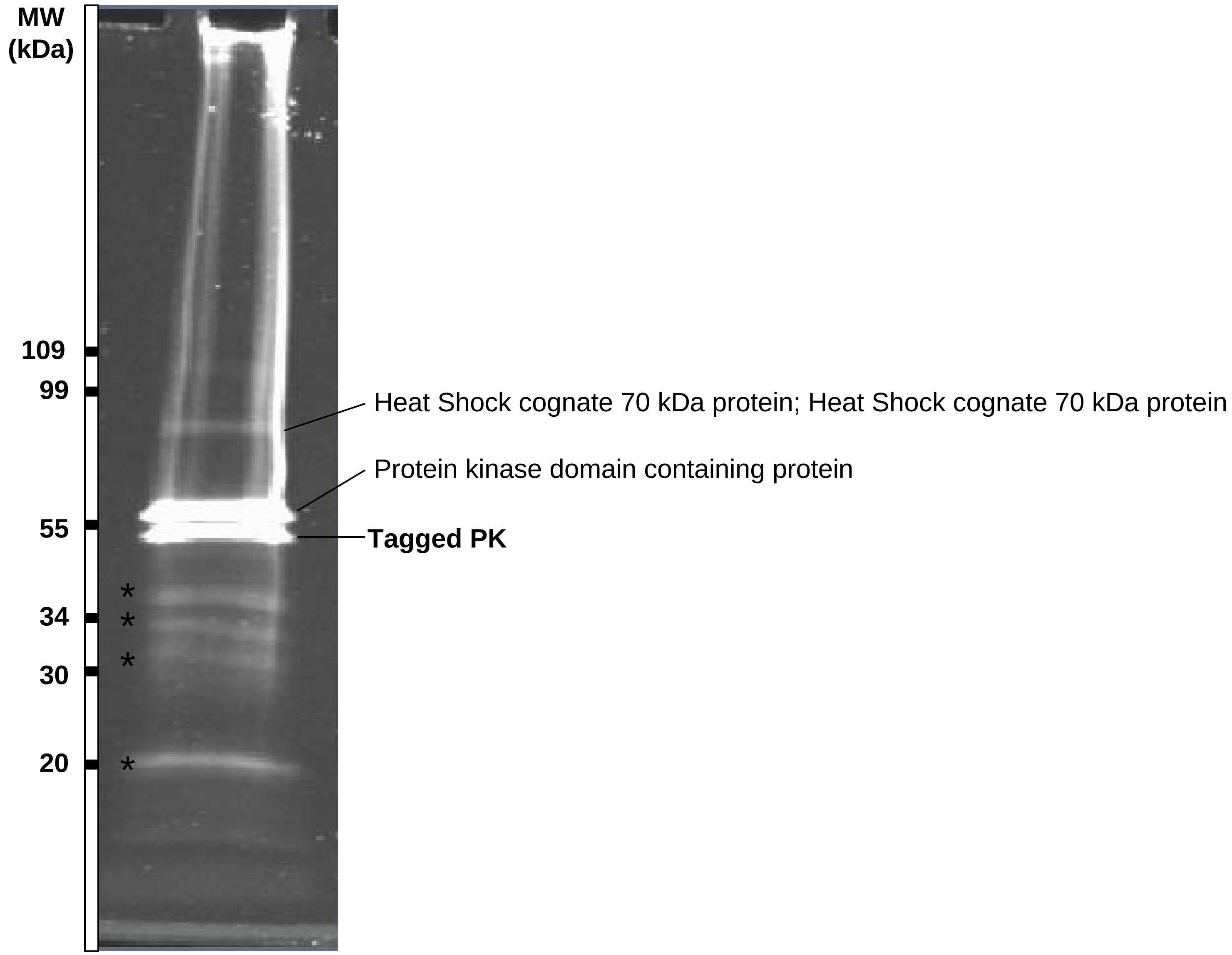

Table 1 # 8: Protein kinase domain containing protein (Os06g50100)

* Contaminant and low scoring tagged protein

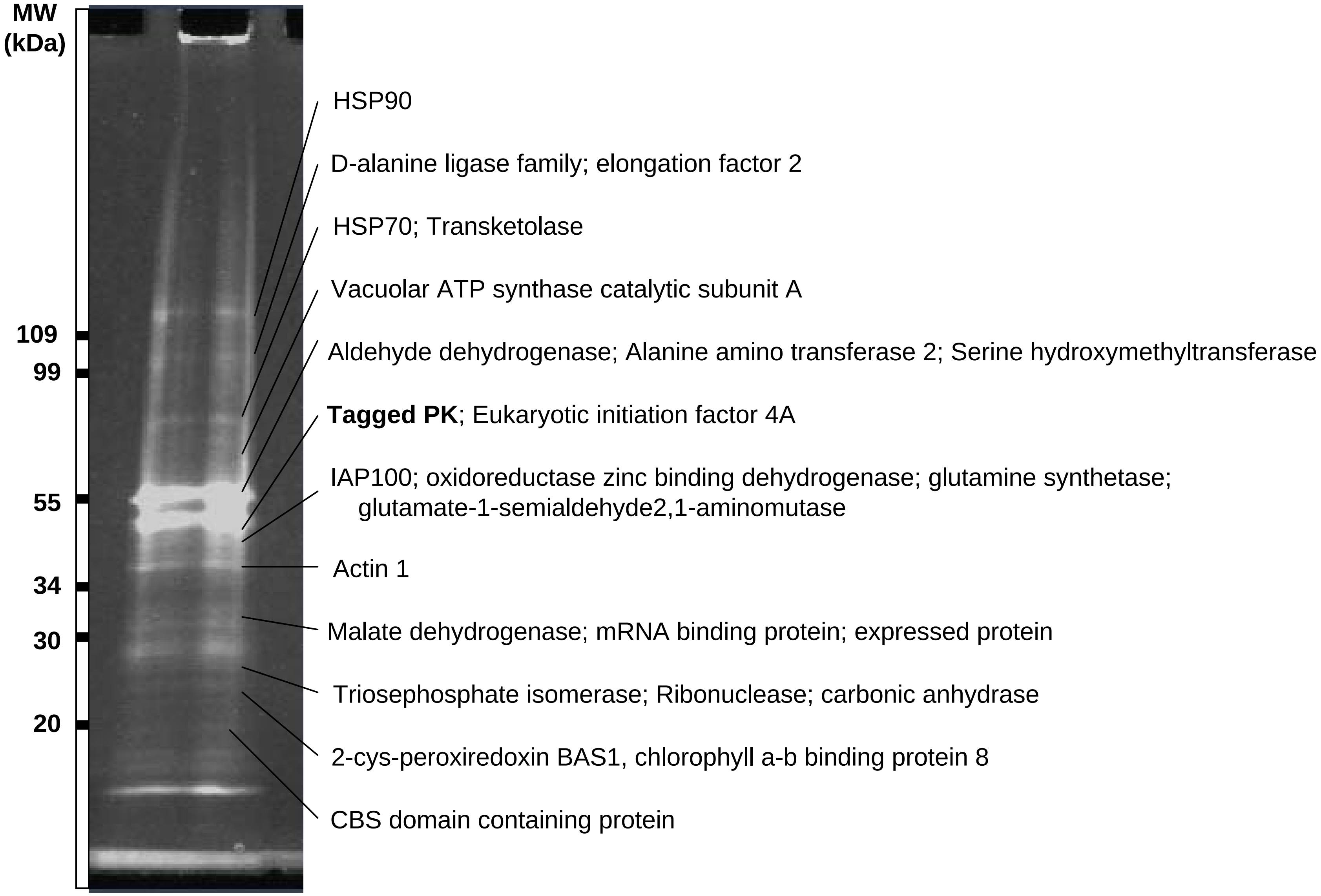

Table 1 # 9: Protein kinase domain containing protein (Os01g67340)

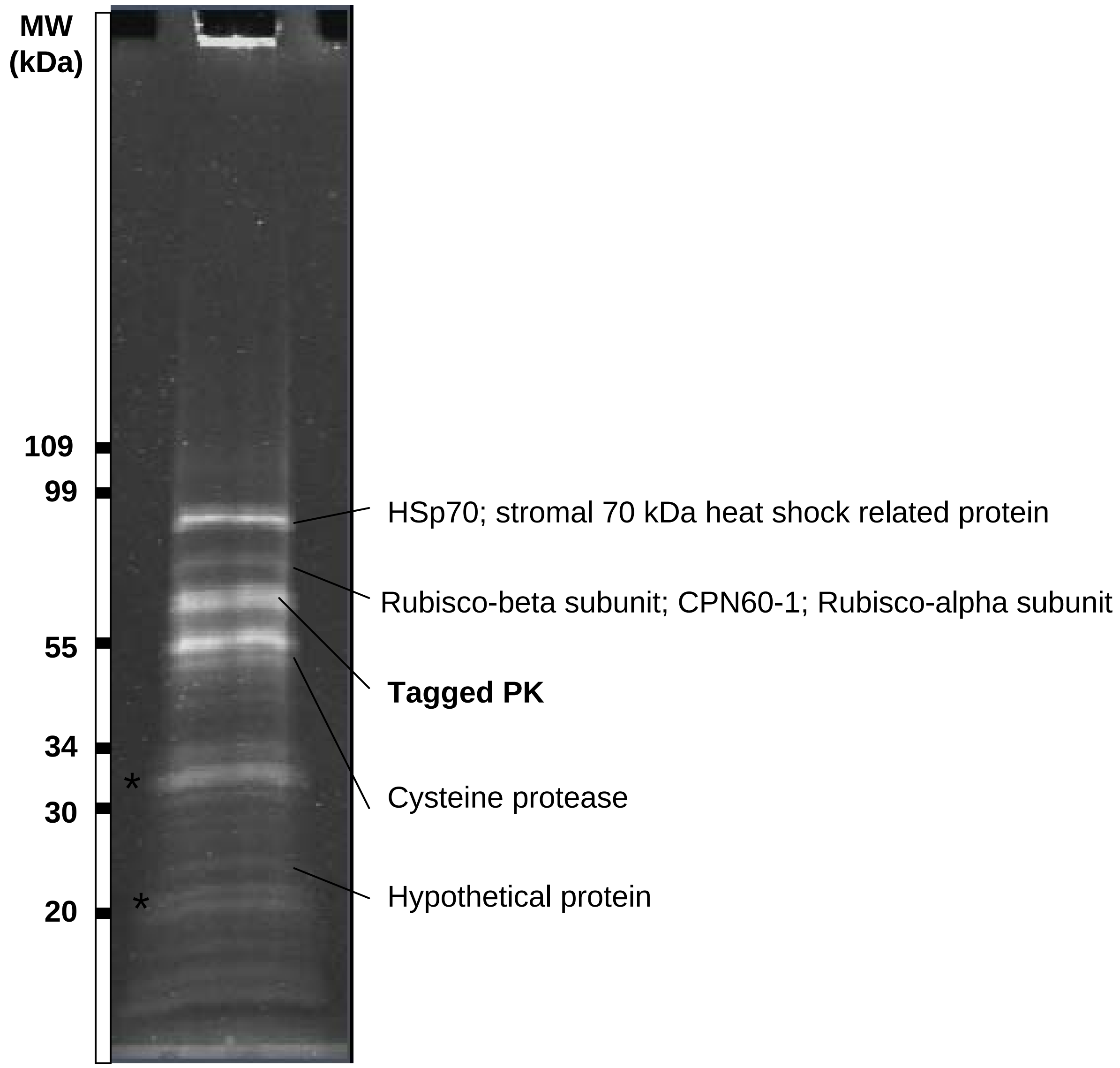

Table 1 # 10: WAK-like kinase (Os03g12470)

* Contaminant and low scoring tagged protein

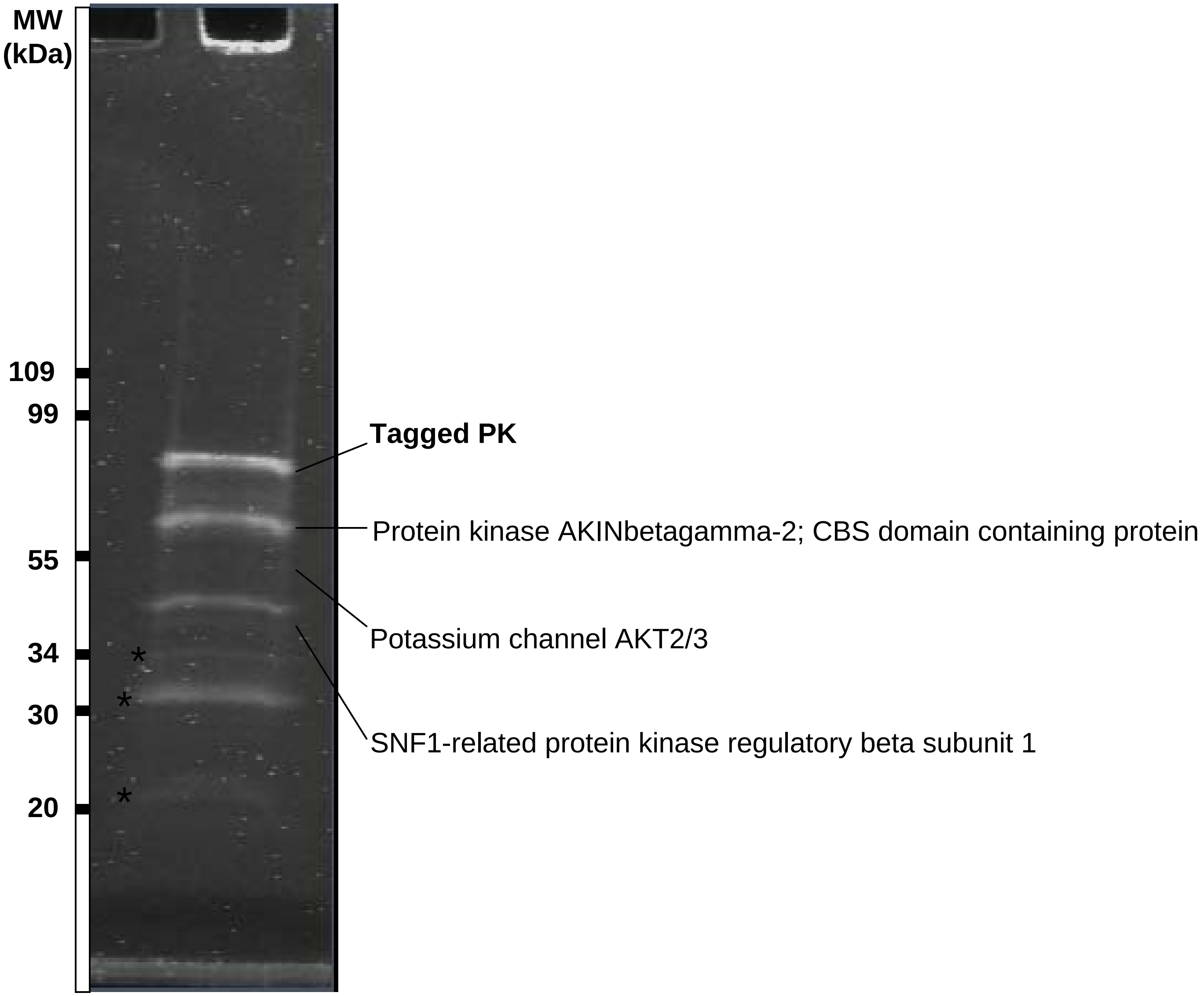

Table 1 # 11: Carbon catabolite derepressing protein kinase (Os08g37800)

* Contaminant and low scoring tagged protein

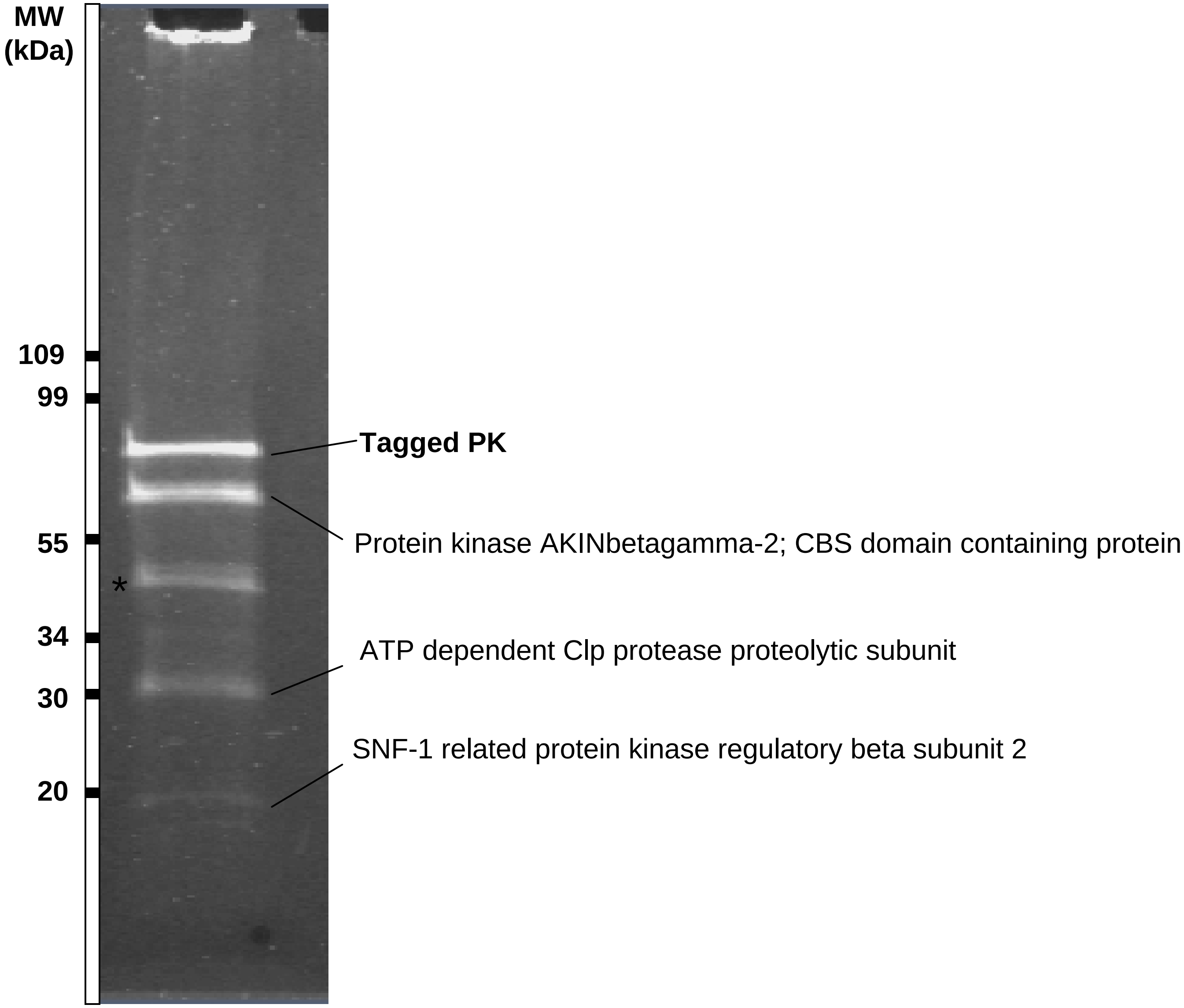

Table 1 # 12: SNF1-related protein kinase (Os05g45420)

* Contaminant and low scoring tagged protein

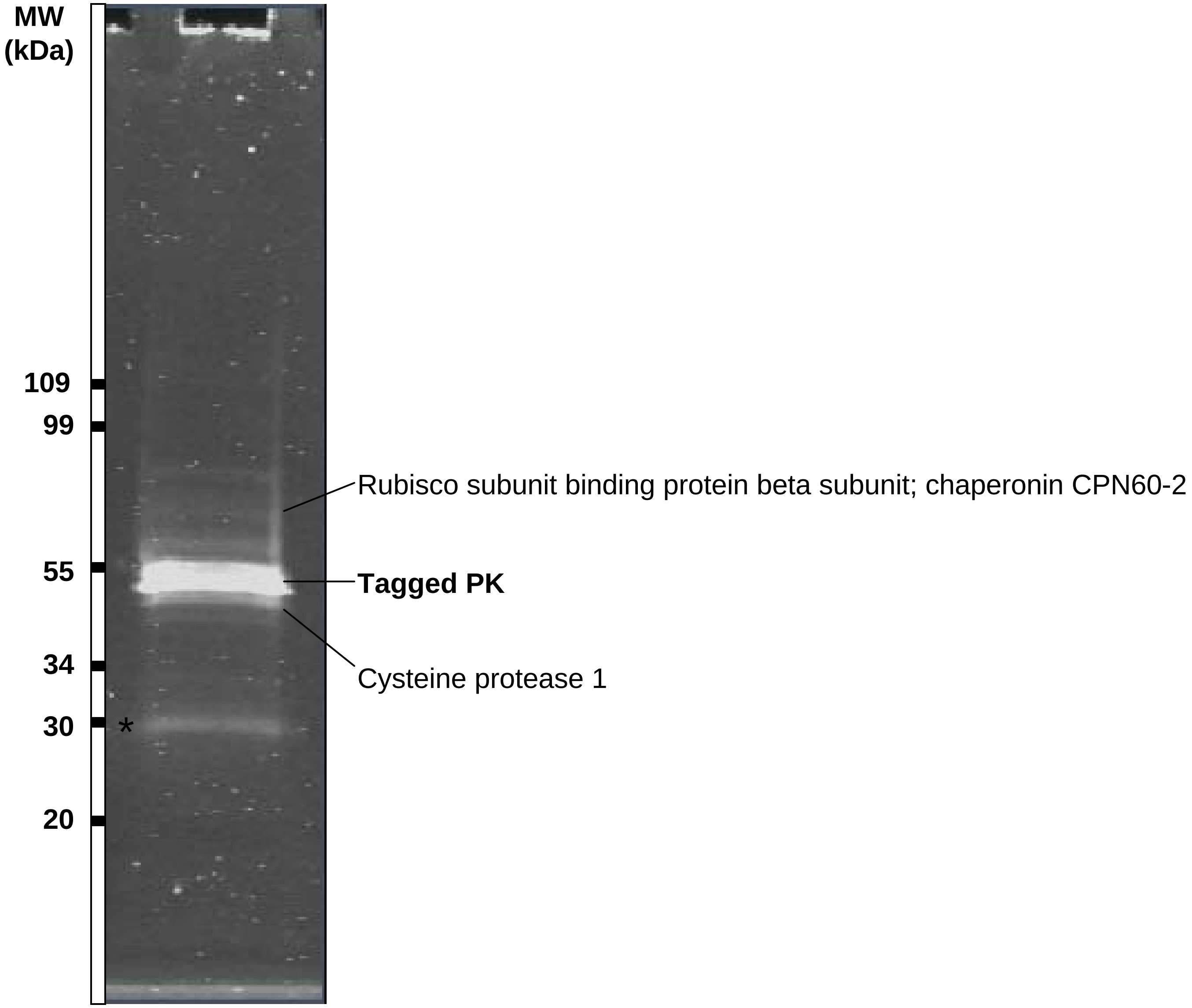

Table 1 # 13: OSMPK14-putative MAPK (Os02g05480)

* Contaminant and low scoring tagged protein
